# Collaborative Innovation Strategy of Supply Chain in the Context of MCU Domestic Substitution : A Differential Game Analysis

**DOI:** 10.1007/s10614-023-10372-9

**Published:** 2023-03-16

**Authors:** Yaxin Wang, Haoyu Wen, ZhongQuan Hu, Yuntao Zhang

**Affiliations:** Xidian University, Xi’an, China

**Keywords:** Collaborative innovation, Supply chain coordination, Differential game, Cost-sharing contracts, Two-part pricing contracts, Domestic substitution

## Abstract

The domestic substitution of the IC (the Integrated Circuit) industry improves economic efficiency and is significant in ensuring national security, which has gradually become an essential strategy for countries worldwide. Based on the background of domestic substitution of integrated circuits, we select a typical component Micro Controller Unit) as the research object, construct a three-level supply chain game model under different scenarios in a dynamic architecture, and analyze the game problem of collaborative innovation of the MCU supply chain. We fully consider the impact of factors such as time, cost and the innovation and collaborative innovation efforts of various supply chain members on the level of domestic substitution. Moreover, we put forward a *two-part pricing + cost-sharing contract* to achieve supply chain coordination. We found that: (1) Collaborative innovation of the supply chain in the centralized decision-making scenario achieves the highest level, followed by the cost-sharing scenario; (2) The *two-part pricing + cost-sharing contract* can help achieve supply chain coordination; (3) The trend of the MCU domestic substitution level with manufacturing cost is *U*-shaped, which means the increase of manufacturing cost may have a positive impact on the process of domestic substitution.

## Introduction

With the increasing competition in the global high-tech industry, the security and vulnerability of the IC supply chain have attracted attention. Since the massive global outbreak of the new coronavirus in 2020, global logistics constraints have led to a chain reaction of rising raw material prices, disruptions in the international supply chain, and a shortage of supplier capacity. The decline in global production capacity has caused a significant impediment to global economic recovery, with the IC (Integrated Circuit) industry being severely impacted. In addition, the recurring epidemics in Malaysia and Taiwan have made it difficult for local factories to resume work, resulting in tight wafer manufacturing capacity and a growing global shortage of chips. As a result of the lack of critical components and delivery delays, downstream integrators in the supply chain, such as Volkswagen, have also been rumoured to stop production. Countries are realizing that having an autonomous and controlled IC supply chain is crucial to securing national information and economic development.

The lack of core technology has led to a mismatch between the manufacturing capacity and the market size of the IC industry in mainland China, with a high dependence on imported IP, design tools, manufacturing equipment, semiconductor materials, etc. In 2020, China’s total chip imports amounted to US$351.5 billion, far exceeding oil as China’s top import. In today’s world, where technology and economic development are inextricably linked, industrial competitiveness is the determining force of a country’s competitive advantage, and the lack of independent control over crucial technologies poses a huge potential threat to national security and economic development. It is imperative to improve technological innovation capabilities, break away from dependence on imported IC products, and achieve domestic replacement of the IC industry.

MCU is the abbreviation for Micro Controller Unit, which is a chip-level computer that combines the frequency and specifications of the central processor with the appropriate reduction of memory, counters, peripheral interfaces such as USB, and even driver circuits in a single chip. In simple terms, an MCU is the equivalent of the brain embedded in electronic products and devices. Currently, the MCU chip market pattern is relatively stable, and the head effect is significant. NXP, Infineon, Renesas electronics, Texas Instruments and other European, American, Japanese and Korean manufacturers have occupied most of the markets for a long time. Although there are more than 40 MCU enterprises in China, the total market share is less than 15%. And only a few enterprises can produce 32-bit general-purpose MCU, while most manufacturers are concentrated in the application fields of household appliances and consumer electronics. In the areas of higher technical and safety requirements, such as automotive electronics and industrial control, most of the technologies, products and application schemes of domestic MCU manufacturers are still immature, which makes downstream enterprises in the supply chain rely heavily on imported products. However, unlike high-end components such as CPUs, MCUs have relatively low requirements for preparation processes. The MCU industry in China is booming, benefiting from the rapid development of China’s Internet of Things and new energy vehicle industries, which has increased the number of connected nodes and the penetration rate of automotive electronics. According to IC Insight research data, China’s MCU market share will grow at a high rate of 9% from 2015 to 2019 and is expected to grow at a CAGR of 9.2% between 2020 and 2025, compared to a CAGR of 7% the global MCU market. In addition, due to the complex international environment and many uncontrollable external factors in recent years, more and more downstream integrators are realizing the importance of a domestic independent supply chain. They are proactively seeking *domestic back-up* or even full replacement products, which brings a rare opportunity to accelerate the MCU localization and replacement process.

Two of the most critical issues in the process of MCU domestic substitution are innovation and the building of an ecosystem. Innovation is the key to sustainable development. Nevertheless, to increase their market share and gain higher profits in the early stages of domestic substitution, some Chinese MCU designers only produce MCUs fully compatible with imported products or even achieve Pin-to-Pin compatibility with imported products. Some companies even sacrificed the performance of their products to downgrade compatibility. This will undoubtedly facilitate the migration of downstream integrators and avoid PCB (Printed Circuit Board) modification costs due to incompatibility. However, it will lead to redundancy in chip design and homogenization of MCU products, which could be more conducive to developing the domestic MCU industry ecosystem. Therefore, to solve the contradiction between innovation and compatibility, companies in the MCU supply chain should invest more in innovation and compatibility to promote the formation of a domestic ecosystem.

When breaking technical barriers and building a domestic ecosystem, in addition to technical problems, collaborative innovation among upstream and downstream enterprises in the supply chain is also very important. Collaborative innovation helps enterprises share costs and risks, acquire new skills and explore commercial markets. Effective R &D cooperation could contribute to the company through product quality, performance and goodwill (Song et al., 2021). In 2021, Infineon, the leading global supplier of MCU solutions, signed an agreement with Hyundai Motor Group, located downstream of the supply chain, to jointly cultivate start-up ecological collaboration and strive to achieve technological breakthroughs in the sensor field through collaborative innovation. In order to reach the high-end quality level, Chinese domestic MCU suppliers should actively cooperate with upstream and downstream enterprises in the supply chain to carry out collaborative innovation, especially in product requirements, product definition, iterative upgrading and application. Suppliers can extract standard customization requirements for application scenarios and specific vertical applications, focusing on the functional integration of higher-performance analogue circuit parts, resulting in a uniquely defined MCU for a market segment.

Therefore, under the background of domestic substitution, this study analyzes the influencing factors of MCU domestic substitution and the incentive coordination mechanism of collaborative innovation within the supply chain and discusses the following problems in detail:

1. What factors influence the optimal strategy of a supply chain member, and how does it vary with these influences? How do the incompatibility issues and manufacturing process mismatches that can arise from designer innovation affect the level of domestic substitution?
2. Under which model is the level of innovation and domestic substitution best?
3. How do cost-sharing contracts affect the optimal strategies of supply chain members? How can cost-sharing contracts be improved to achieve coordination of supply chain members’ strategies?

The remainder of the paper is organised as follows. Section 2 reviews the relevant literature. In Sect. 3, the problem description and parameter assumptions are presented. Section 4 constructs a collaborative innovation model in different contexts and finds the equilibrium solution. Section 5 compares the optimal decisions of supply chain members in different situations and designs supply chain coordination. Section 6 presents the results of the numerical analysis. Section 7 summarises the findings and identifies future research directions.

## Literature Review

In this section, we first review the related literature, including collaborative innovation, differential games, supply chain coordination, and domestic research in the Chinese IC industry. Then, we summarize the research gap between this literature and our study.

### Collaborative Innovation in the Supply Chain

Supply chain innovation has been widely recognized as an important factor in improving supply chain performance, and collaborative innovation can bring several economic, environmental and social benefits (Krishnan et al., 2021). Li et al. (2021) constructed a game model for the evolution of enterprises in the supply chain among strategic emerging industries. They concluded that collaborative innovation among supply chain enterprises is a meaningful way to accelerate the extension of the industrial chain to the high end. Hong and Guo (2019) studied the issue of collaborative innovation and product diffusion of green technologies in a two-tier green product supply chain consisting of manufacturers and retailers and showed that social welfare increases with the level of collaboration of supply chain members. Huang et al. (2020) have constructed a discussion on how to carry out collaborative innovation in the context of frequent trade frictions in complex equipment, which provides a certain reference for the strategic choice of supply chain members. Shen et al. (2021) studied supply chain collaboration when suppliers and manufacturers work together on a product that contains several innovative elements. They argue that the optimal profits of supply chain members are greater in the case of collaborative innovation than in the case of single-product innovation when the market size is large enough.

In summary, research on collaborative innovation in supply chains has yielded rich results, laying a solid theoretical foundation for the research in this paper. However, most existing studies focus on the impact of technological innovation inputs on supply chain performance. Due to the particular characteristics of the IC industry, compatibility and process improvement in the supply chain should also be considered in the collaborative innovation game model. Moreover, most of the above studies on collaborative innovation in the supply chain are based on a static framework rather than considering the impact of the time factor on collaborative innovation in the supply chain. Unlike the above literature, the innovation activities in the IC industry are a long-term, dynamic process that requires continuous technological innovation and cooperation. Therefore, it would be more practical to introduce the time factor to study the MCU supply chain’s long-term dynamic, collaborative innovation activities and coordination mechanism based on the existing studies.

### Differential Games

The differential game, one of the essential dynamic game models, is an academic tool to deal with the problem of adversarial conflict, competition and cooperation between parties in the supply chain in continuous time (Zhao et al., 2017). Lu et al. (2019) studied the dynamic game problem of advertising and supply chain coordination between manufacturers and retailers. Wang et al. (2021) discussed the impact of consumers’ low-carbon preference on the collaborative emission reduction strategies of manufacturers and suppliers in three different situations. Li (2020) investigated the effects of credit support, government subsidies, and internal cost subsidies on the socially responsible behaviour of supply chain member firms by constructing a differential game model consisting of a single manufacturer and a single retailer, in which the profit sharing coefficient determines both profits. However, the studies mentioned above use the marginal profit of the supply chain members and the product of market demand to represent the profit when building the differential game model without considering the influence of price and manufacturing cost on the game outcome. Moreover, MCU’s manufacturing cost and selling price have significantly increased due to the lack of capacity in the IC supply chain in recent years. Studying the impact of price and cost on MCU collaborative innovation is necessary and practical.

Guan et al. (2020) introduced price as the control variable in the differential game model to determine the optimal quality improvement level and advertising effort level of the supply chain under the condition that the supply chain members pay extra attention to fairness. By developing a Stackelberg differential game model between manufacturers and retailers, Song et al. (2021) investigates the relationship between the goodwill of a product and its degree of innovation, as well as the optimal dynamic pricing strategy in the supply chain. Based on the above two studies, we consider the impact of price and non-price factors of MCUs on supply chain performance in a differential game model, drawing on the demand separation multiplication method mentioned in the study of El Ouardighi (2014).

Other than that, the above studies are based on a secondary supply chain. Due to the unique nature of the MCU production and manufacturing process, we need to establish a three-tier supply chain consisting of manufacturers, designers and integrators to carry out the research. The designer is responsible for designing the innovative MCU, which is then handed over to the manufacturer for engineering batch flow and mass production of the finished product. The designer sells the MCU delivered by the manufacturer to the integrator, who then integrates the MCU to produce a circuit module that can be used in a particular scenario (e.g. a control system for a smart home). This combination of MCU design and production process features is more relevant.

### Supply Chain Coordination

Many scholars have studied a variety of supply chain coordination contracts, such as revenue-sharing contracts, cost-sharing contracts, resale contracts and two pricing contracts (Avinadav et al., 2021; Lu et al., 2019; Liu et al., 2020; Cheng et al., 2020; Fan et al., 2020; Ni et al., 2021), to help managers make better supply chain coordination decisions. Among them, He et al. (2020) considered corporate social responsibility and investigated the optimal service level, carbon reduction, and optimal decision of advertising efforts of supply chain members in conducting low-carbon service and advertising cooperation, and compared the effects of different cost-sharing contracts on supply chain performance.In addition, cost-sharing contracts are an effective means to encourage collaborative innovation in areas such as green supply chain (Wang et al., 2021), desertification control(Sun and Tan, 2021) and big data marketing (Xiang and Xu, 2020). However, Lu et al. (2017) studied the cooperation between manufacturers and retailers in promotions and advertising and concluded that cost-sharing could increase the revenue of each party but not achieve supply chain coordination. Similarly, Wang et al. (2021) argue that supply chain members’ efforts and market demand are higher under centralized decision making than under decentralized decision making, and that cost-sharing and wholesale price contracts cannot coordinate the supply chain. Therefore, they designed a two-part pricing contract to coordinate the strategies of manufacturers and retailers under decentralized decision making.argue that supply chain members’ efforts and market demand are higher under centralized decision-making than under decentralized decision-making and that cost-sharing and wholesale price contracts cannot coordinate the supply chain. Liu et al. (2016) studied the supply chain synergy problem when downstream firms in the supply chain have fair preferences. They concluded that the coordination of pricing contracts between two parts of the supply chain could be reached only when the fair preferences of manufacturers reach a certain critical value.

In summary, most of the above studies use a single contract to improve the strategies of supply chain members. Although these contracts can improve supply chain performance, the optimal decisions of supply chain members are still lower than when decisions are centralized. To achieve supply chain coordination, this study combines a two-part pricing contract with a cost-sharing contract to ensure a fair distribution of benefits while improving the collaborative innovation efforts of non-dominant firms in the game.

### Localization of the Chinese IC Industry

The IC industry is a strategic, fundamental and pioneering industry for national economic and social development. Promoting the domestic substitution level of the IC industry is not only of economic significance but also an inevitable way to ensure national security and the autonomy and control of critical industries. The IC industry development has now risen to the level of a national strategy(Kose and Friedman, 2012). Zhou (2018) analyzed the development history of the integrated circuit industry in the United States, Japan, and Korea based on detailed research and proposed recommendations for the development of China’s IC industry. By analyzing the scene specificity of power chips, combing the design architecture of power chips and discussing the application scenarios and research focuses of power chips, Ma et al. (2021) concluded that breaking through the key technology bottleneck is the key to solving the chip autonomy and controllability and achieving domestic substitution. However, these studies are mainly based on analysing and summarising existing literature and information. They have not yet established a scientific game model to propose and argue for the development of the IC industry. However, the above research is mainly based on the analysis and summary of the existing literature and information and has not been demonstrated by establishing a scientific game model. Based on these studies and the trend of domestic substitution in China’s IC industry, this paper selects representative components MCU as the research object. It establishes a collaborative innovation game model of three companies (manufacturer, designer and integrator) in the MCU supply chain.

Although the above literature has studied the issue of collaborative supply chain innovation in various contexts, there are specific gaps: (1) Previous studies have mainly used static game approaches without considering changes in supply chain members’ strategies, and relevant state variables over time. (2) Most current studies on IC domestic substitution are subjective comments based on literature and industry development rather than conducting qualitative research by establishing game models. (3) Nowadays, scholars usually use a single contract to improve the optimal decision of supply chain members under decentralized decision-making. However, the results show that a single contract is challenging to achieve supply chain coordination.

To summarize, this study will study the problem of MCU domestic substitution by establishing a differential game model of collaborative innovation in a three-level supply chain consisting of upstream manufacturers, MCU chip designers and downstream integrators. The optimal strategy of collaborative innovation will be analyzed by comparing the optimal effort and profit of the supply chain members under different situations. Finally, a *cost-sharing + two pricing* contract is designed to achieve supply chain coordination. Compared with the existing literature, the contributions of this paper are:

1. Bridging the gap in quantitative research in the domestic replacement of integrated circuits using the differential game methodology.
2. In a dynamic framework, we investigate the optimal decisions of supply chain members for collaborative innovation and the associated state variables over time.
3. This study combines a cost-sharing contract with a two-part pricing contract to achieve supply chain coordination.
4. We study the impact of the significant cost increase of design and manufacturing on the local substitution process under the current global IC capacity shortage. Through comparative analysis and numerical simulations, we have obtained an exciting conclusion: MCU manufacturing cost increase may reduce the level of domestic substitution in the initial stage. However, the domestic substitution level will increase with time and continuous cost increases.

## Problem Description and Model Assumption

**Fig. 1:**
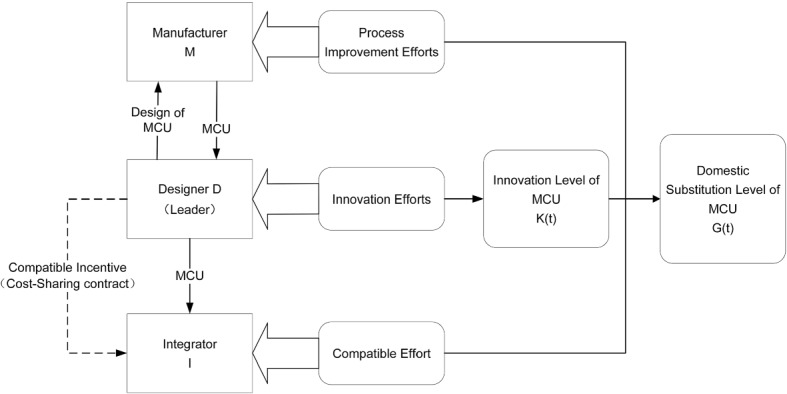
Model Structure

We consider a three-tier supply chain consisting of a wafer manufacturer M (hereinafter referred to as the manufacturer), an MCU designer D (hereinafter referred to as the designer), and a downstream MCU application integrator I (hereinafter referred to as the integrator).Among them, MCU designers, as the leader of the game, are responsible for designing innovative and market-competitive MCU products and need to invest in innovation efforts ${E_D}\left( t \right)$ .After the design is completed, the designer sends the product manufacturing requirements to the upstream manufacturer for flow and production. The manufacturer M need to invest in process improvement efforts ${E_M}\left( t \right)$ in order to better manufacture according to the design. The manufacturer M delivers the MCU to the designer D after successful completion of engineering lot flow and mass production of the finished product, and charges ${{{\omega }}_1}\left( t \right)$ .The designer D sells the MCU at price ${{{\omega }}_2}\left( t \right)$ to the integrator I ,who needs to invest compatible effort ${E_I}\left( t \right)$ in order to use the MCU for further production. Figure 1 shows the model structure.

According to the characteristics of manufacturing in the IC industry, the cost of MCU innovation is determined by the degree of innovation because of the practical problems such as uneconomical production scale, scarcity of production resources or difficulty in expanding production. The higher the degree of innovation, the faster the rate of cost increase will be. The cost of innovation for upstream manufacturers ,${\mathrm{{C}}_D}\left( {{E_D}\left( t \right) } \right)$,is therefore a convex function of the level of innovation effort.

1 $$\begin{aligned} {\mathrm{{C}}_D}\left( {{E_D}\left( t \right) } \right) = \frac{1}{2}{\gamma _D}{E_D}^2\left( t \right) \; \end{aligned}$$

where ${\gamma _D} > 0$ is the manufacturer’s innovation cost factor. This form of cost function captures the law of diminishing returns and is commonly used in the literature (Krishnan et al., 2019; Wang et al., 2019; Zhou et al., 2016; Ouardighi et al., 2019).

As mentioned by Zhang et al. (2020), innovation can lead to mismatch costs for other members in the supply chain. In the MCU co-innovation process, also breakthrough innovations by designers can cause mismatch costs for downstream integrators, known as the incompatibility problem. The integrator must invest more effort in hardware and software compatibility to overcome it. This paper assumes that the downstream integrator’s compatible effort costs can be expressed as:

2 $$\begin{aligned}{} & {} {\mathrm{{C}}_I}\left( {{E_I}\left( t \right) } \right) = \frac{1}{2}{\gamma _I}{E_I}^2\left( t \right) \; \end{aligned}$$

3 $$\begin{aligned}{} & {} {\mathrm{{C}}_M}\left( {{E_M}\left( t \right) } \right) = \frac{1}{2}{\gamma _M}{E_M}^2\left( t \right) \; \end{aligned}$$

Among them, ${\gamma _I} > 0$ and ${\gamma _M} > 0$ are the compatible cost coefficient and process improvement cost coefficient of manufacturers and integrators respectively.

Due to the complexity of the IC design and production process, MCU innovation is achieved over time. The innovation level of MCU is not only related to the designer’s innovation efforts but also has a specific decay rate with time for technological progress and other reasons, which has the characteristics of dynamic change (Liu et al., 2020). Therefore, we assume that ${K\left( t \right) }$ represents the innovation level of MCU at time t, which is influenced by the degree of innovation efforts of MCU designers and will continue to improve with the improvement of MCU product innovation level. Therefore, the dynamic process of MCU innovation over time can be expressed by the following differential equation:

4 $$\begin{aligned} \begin{array}{*{20}{c}} {\mathrm{{\dot{K}}}\left( \mathrm{{t}} \right) = \frac{{dK\left( t \right) }}{{dt}} = {\lambda _D}{E_D}\left( t \right) - \delta K\left( t \right) } \end{array}\; \end{aligned}$$

where $\mathrm{{K}}\left( 0 \right) = {\mathrm{{\textrm{K}}}_0} \ge 0$. ${\lambda _D}$ indicates the impact of upstream manufacturers’ innovation efforts on MCU product innovation level, that is, innovation impact factor. In the process of MCU design and development, the technology will be updated and evolved, so $\delta > 0$ indicates the decline rate of the innovative technology in the development process (Dawid et al., 2020).

In this paper, the level of innovation , and the degree of synergistic innovation cooperation between integrators and manufacturers (i.e. the level of process improvement efforts by integrators and the level of compatibility efforts by manufacturers) all have a positive impact on the level of domestic substitution. And as Moore’s theorem continues to approach its limits and competition between countries and firms develops, there is a natural decay in the level of technology in emerging strategic industries (Zhao et al., 2017). The dynamic pattern of change in the level of domestic substitution is similar to the rationale behind the dynamic change in the level of goodwill for innovative products in the study of Yao et al. (2021); Biancardi et al. (2021). Referring to the classical model of (Nerlove and Arrow, 1962), we describe the dynamics of the level of domestic substitution over time as a differential equation:

5 $$\begin{aligned} \begin{array}{*{20}{c}} {\mathrm{{\dot{G}}}\left( \mathrm{{t}} \right) = \frac{{dG\left( t \right) }}{{dt}} = {\lambda _I}{E_I}\left( t \right) + {\lambda _M}{E_M}\left( t \right) + \varepsilon K\left( t \right) - \theta G\left( t \right) } \end{array}\; \end{aligned}$$

where $\mathrm{{G}}\left( 0 \right) = {\mathrm{{\mathrm{}}}_0} \ge 0$. ${\lambda _I}$ indicates the impact of an integrator’s compatibility efforts on the level of domestic substitution of an end product, that is, the compatibility factors. ${\lambda _M}$ indicates factors influencing process improvement. $\mathrm{{\varepsilon \;}}$ indicates the influence factor of innovation level on domestic substitution level.$\theta$ indicates the rate of decline of domestic substitution levels during R &D.

Demand is inherently dynamic, and both the level of MCU innovation and the level of domestic substitution positively influence the market demand for the product. Therefore, referring to the study of El Ouardighi (2014) that divides the factors that have an impact on market demand into price and non-price factors, and that these two factors can have an impact on demand by separating into phases, we write the dynamic demand as:

6 $$\begin{aligned} \begin{array}{*{20}{c}} {D\left( \mathrm{{t}} \right) = \left( {\alpha - {{{\beta }}_P}P\left( t \right) } \right) \left( {{{{\beta }}_\mathrm{{K}}}K\left( t \right) + {{{\beta }}_G}G\left( t \right) } \right) } \end{array}\; \end{aligned}$$

where ${D\left( t \right) }$ is the demand for the product at moment t. $\mathrm{{\alpha }}>0$, ${{{\beta }}_P}>0$ , ${{{\beta }}_{{K}}}>0$ , ${{{\beta }}_G}>0{{\;}}$ are the degree of influence of price, MCU innovation level and domestic substitution level on market demand, respectively.

We assume that at any given moment the supply chain members have the same discount factor ${{\;\rho }}\left( {{\rho }} > 0\right)$ as Wang and Wang (2021) and Wang et al. (2021) do in their research.

For writing convenience, t will be omitted in the following.

The model parameters are described in Table 1.

**Table 1:** Notations and definitions

Parameters	Definitions	Parameters	Definitions
${{E}_{D}},{{E}_{I}},{{E}_{M}}$	Designer’s innovation investment , Integrator’s compatibility investment , Manufacturer’s process improvement investment	$\text {p}$	Selling price of integrators to downstream markets
${{\gamma }_{D}},{{\gamma }_{I}},{{\gamma }_{M}}$	Influence coefficient of cost on the innovation effort, compatibility effort and process improvement effort	$\mathrm{{\alpha }}$	Market size
${{\text {C}}_{D}},{{\text {C}}_{I}},{{\text {C}}_{M}}$	Collaborative innovation costs for designers , integrators and manufacturers	$\text {D}$	Demand of downstream markets
${{\beta }_{P}},{{\beta }_{K}},{{\beta }_{G}}$	Influence coefficient of price, level of innovation and level of domestic substitution on market demand	$\text {K}$	The level of innovation
${{\lambda }_{D}}$	Influence coefficient of innovation effect on the level of innovation	$\text {G}$	The level of domestic substitution
${{\lambda }_{I}}$	Influence coefficient of compatibility effort on the level of domestic substitution	$\varepsilon$	Influence coefficient of the level of innovation on the level of domestic substitution
${{\lambda }_{I}}$	Influence coefficient of process improvement effort on the level of domestic substitution	$\theta$	Natural decay rate of the domestic substitution
${{\text { }\!\!\omega \!\!\text { }}_{1}}$	The wholesale price paid by the designer to the manufacturer	$\text { }\!\!\delta \!\!\text { }$	Natural decay rate of the innovation level
${{\text { }\!\!\omega \!\!\text { }}_{2}}$	The wholesale price paid by the integrator to the designer	$\rho$	Discount rate
$\text { }\!\!\sigma \!\!\text { }$	Cost-sharing factor		

## Model Formulation and Analysis

In this section, we discuss the optimal strategies of supply chain members in the case of non-cooperative mode, cost-Sharing mode and cooperative mode, respectively.

### Decentralized Decision-making without Cost-sharing Contract (Case n)

In the non-cooperative mode,the manufacturer determines its process improvement efforts ${\mathrm{{E}}_M}$ and wholesale price ${\mathrm{{\omega }}_1}$. Designers decide their innovation efforts ${\mathrm{{E}}_D}$ and wholesale price ${\mathrm{{\omega }}_2}$, Integrator decides compatibility efforts ${\mathrm{{E}}_I}$ and selling price $\mathrm{{p}}$. Supply chain members all make their decisions independently at the same time to maximize their profits. The decision-making issues of three parties are:

7 $$\begin{aligned} \mathrm{{\Pi }}_M^n= & {} \mathop \smallint \nolimits _0^\infty {e^{ - \rho t}}[\left( {{{{\omega }}_1} - c} \right) \left( {\alpha - {{{\beta }}_\mathrm{{P}}}p} \right) ({{{\beta }}_{\mathrm{K}}}K + {{{\beta }}_G}G) - \frac{1}{2}{\gamma _M}{{\left( {E_M^n} \right) }^2}]dt\; \end{aligned}$$

8 $$\begin{aligned} \mathrm{{\Pi }}_D^n= & {} \mathop \smallint \nolimits _0^\infty {e^{ - \rho t}}[\left( {{{{\omega }}_2} - {{{\omega }}_1}} \right) \left( {\alpha - {{{\beta }}_\mathrm{{P}}}p} \right) ({{{\beta }}_\mathrm{{K}}}K + {{{\beta }}_G}G) - \frac{1}{2}{\gamma _D}{{\left( {E_D^c} \right) }^2}]dt\; \end{aligned}$$

9 $$\begin{aligned} \mathrm{{\Pi }}_I^n= & {} \mathop \smallint \nolimits _0^\infty {e^{ - \rho t}}[\left( {p - {{{\omega }}_2}} \right) \left( {\alpha - {{{\beta }}_\mathrm{{P}}}p} \right) ({{{\beta }}_\mathrm{{K}}}K + {{{\beta }}_G}G) - \frac{1}{2}{\gamma _I}{{\left( {E_I^n} \right) }^2}]dt\; \end{aligned}$$

#### Proposition 1

In the situation of decentralized decision-making without cost-sharing contracts, the equilibrium results of the differential game among manufacturers, designers and integrators are as follows:

(1) The optimal process improvement efforts of the manufacturer is:

10 $$\begin{aligned} {E_{M}^{n}}^* =\frac{{{\text { }\!\!\Delta \!\!\text { }}_{1}}{{\text { }\!\!\beta \!\!\text { }}_{\text {G}}}{{\lambda }_{M}}}{\left( \rho +\theta \right) {{\gamma }_{M}}}\; \end{aligned}$$

The optimal innovation efforts of the designer is:

11 $$\begin{aligned} E{{_{D}^{n}}^{*}}=\frac{{{\text { }\!\!\Delta \!\!\text { }}_{2}}\left[ {{\text { }\!\!\beta \!\!\text { }}_{\text {k}}}\left( \rho +\theta \right) +{{\text { }\!\!\beta \!\!\text { }}_{\text {G}}}\text { }\!\!\varepsilon \!\!\text { } \right] {{\lambda }_{D}}}{\left( \rho +\delta \right) \left( \rho +\theta \right) {{\gamma }_{D}}}\; \end{aligned}$$

The optimal compatibility efforts of the integrator is:

12 $$\begin{aligned} {E_{I}^{n}}^* =\frac{{{\text { }\!\!\Delta \!\!\text { }}_{3}}{{\text { }\!\!\beta \!\!\text { }}_{\text {G}}}{{\lambda }_{I}}}{\left( \rho +\theta \right) {{\gamma }_{I}}}\; \end{aligned}$$

where $${\mathrm{{\Delta }}_1} = \left( {{{{\omega }}_1}^* - c} \right) \left( {{{\alpha }} - {{{\beta }}_\mathrm{{P}}}\mathrm{{p}}} \right) = \frac{{{{\left( {{{\alpha }} - {{{\beta }}_\mathrm{{P}}}\mathrm{{c}}} \right) }^2}}}{{16{{{\beta }}_\mathrm{{P}}}}}$$,$${\mathrm{{\Delta }}_2} = ({{{\omega }}_2}^* - {{{\omega }}_1}^*)\left( {{{\alpha }} - {{{\beta }}_\mathrm{{P}}}\mathrm{{p}}} \right) = \frac{{{{\left( {{{\alpha }} - {{{\beta }}_\mathrm{{P}}}\mathrm{{c}}} \right) }^2}}}{{32{{{\beta }}_\mathrm{{P}}}}}$$,$${\mathrm{{\Delta }}_3} = \left( {{{{p}}^*} - {{{\omega }}_2}^*} \right) \left( {{{\alpha }} - {{{\beta }}_\mathrm{{P}}}\mathrm{{p}}} \right) = \frac{{{{\left( {{{\alpha }} - {{{\beta }}_\mathrm{{P}}}\mathrm{{c}}} \right) }^2}}}{{64{{{\beta }}_\mathrm{{P}}}}}$$.

It can be seen that the degree of collaborative innovation effort of supply chain member firms increases with the influence coefficient of their collaborative efforts on the level of innovation and domestic substitution in the supply chain and decreases with the cost coefficient.

(2) The optimal price decisions are as follows:

13 $$\begin{aligned} {{{{\omega }}_1}^* = \frac{{{{\alpha }} + {{{\beta }}_\mathrm{{P}}}{{c}}}}{{2{{{\beta }}_{{P}}}}}}\; \end{aligned}$$

The optimal innovation efforts of the designer is:

14 $$\begin{aligned} {{{{\omega }}_2}^* = \frac{{3{{\alpha }} + {{{\beta }}_\mathrm{{P}}}{{c}}}}{{4{{{\beta }}_\mathrm{{P}}}}}}\; \end{aligned}$$

The optimal compatibility efforts of the integrator is:

15 $$\begin{aligned} {{{{p}}^*} = \frac{{7{{\alpha }} + {{{\beta }}_\mathrm{{P}}}{{c}}}}{{8{{{\beta }}_{{P}}}}}}\; \end{aligned}$$

The optimal pricing for each supply chain member increase with the potential market and manufacturing cost and decrease with the price influencing factors.

(3) The optimal profits of each member in the supply chain are as follows:

16 $$\begin{aligned}   {\text{W}}_{M}^{{n*}}  =  & \,\,\frac{{\Delta _{1} [\beta _{{\text{k}}} \left( {\rho  + \theta } \right) + \beta _{{\text{G}}} \varepsilon ]}}{{\left( {\rho  + \delta } \right)\left( {\rho  + \theta } \right)}}K + \frac{{\Delta _{1} \beta _{{\text{G}}} }}{{\left( {\rho  + \theta } \right)}}G \\     +  & \,\,\frac{1}{\rho }\left[ {\frac{{\Delta _{1} ^{2} \beta _{{\text{G}}} ^{2} \lambda _{M} ^{2} }}{{2\gamma _{M} \left( {\rho  + \theta } \right)^{2} }} + \frac{{\Delta _{1} \Delta _{2} [\beta _{{\text{k}}} \left( {\rho  + \theta } \right) + \beta _{{\text{G}}} \varepsilon ]^{2} \lambda _{D} ^{2} }}{{\gamma _{D} \left( {\rho  + \delta } \right)^{2} \left( {\rho  + \theta } \right)^{2} }} + \frac{{\Delta _{1} \Delta _{3} \beta _{{\text{G}}} ^{2} \lambda _{I} ^{2} }}{{\gamma _{I} \left( {\rho  + \theta } \right)^{2} }}} \right]\; \\  \end{aligned} $$

17 $$ \begin{aligned}   W_{D}^{{n*}}  =  & \,\frac{{\Delta _{2} \left[ {\beta _{{\text{k}}} \left( {\rho  + \theta } \right) + \beta _{{\text{G}}} \varepsilon } \right]}}{{\left( {\rho  + \delta } \right)\left( {\rho  + \theta } \right)}}K + \frac{{\Delta _{2} \beta _{{\text{G}}} }}{{\left( {\rho  + \theta } \right)}}G \\     +  & \,\,\frac{1}{\rho }\left[ {\frac{{\Delta _{2} \Delta _{1} \beta _{{\text{G}}} ^{2} \lambda _{M} ^{2} }}{{\gamma _{M} \left( {\rho  + \theta } \right)^{2} }} + \frac{{\Delta _{2} ^{2} [\beta _{{\text{k}}} \left( {\rho  + \theta } \right) + \beta _{{\text{G}}} \varepsilon ]^{2} \lambda _{D} ^{2} }}{{2\gamma _{D} \left( {\rho  + \delta } \right)^{2} \left( {\rho  + \theta } \right)^{2} }} + \frac{{\Delta _{2} \Delta _{3} \beta _{{\text{G}}} ^{2} \lambda _{I} ^{2} }}{{\gamma _{I} \left( {\rho  + \theta } \right)^{2} }}} \right] \\  \end{aligned}  $$

18 $$ \begin{aligned}   {\text{W}}_{l}^{{n*}}  =  & \,\frac{{\Delta _{3} \left[ {\beta _{{\text{k}}} \left( {\rho  + \theta } \right) + \beta _{{\text{G}}} \varepsilon } \right]}}{{\left( {\rho  + \delta } \right)\left( {\rho  + \theta } \right)}}K + \frac{{\Delta _{3} \beta _{{\text{G}}} }}{{\left( {\rho  + \theta } \right)}}G \\     +  & \,\,\frac{1}{\rho }\left[ {\frac{{\Delta _{3} \Delta _{1} \beta _{{\text{G}}} ^{2} \lambda _{M} ^{2} }}{{\gamma _{M} \left( {\rho  + \theta } \right)^{2} }} + \frac{{\Delta _{2} \Delta _{3} [\beta _{{\text{k}}} \left( {\rho  + \theta } \right) + \beta _{{\text{G}}} \varepsilon ]^{2} \lambda _{D} ^{2} }}{{\gamma _{D} \left( {\rho  + \delta } \right)^{2} \left( {\rho  + \theta } \right)^{2} }} + \frac{{\Delta _{3} ^{2} \beta _{{\text{G}}} ^{2} \lambda _{I} ^{2} }}{{2\gamma _{I} \left( {\rho  + \theta } \right)^{2} }}} \right] \\  \end{aligned}  $$

(4) The optimal trajectory of the MCU innovation level is:

19 $$\begin{aligned} {{\mathrm{{K}}^{n*}} = \left[ {{\mathrm{{K}}_0} - \mathrm{{K}}_\infty ^n} \right] {e^{ - \delta t}} + \mathrm{{K}}_\infty ^n}\; \end{aligned}$$

where $$\mathrm{{K}}_\infty ^n = \frac{1}{\delta }\left( {\frac{{{\mathrm{{\Delta }}_2}[{\beta _K}\left( {\rho + \theta } \right) + {\beta _G}\varepsilon ]{\lambda _D}^2}}{{{\gamma _D}\left( {\rho + \delta } \right) \left( {\rho + \theta } \right) }}} \right) $$.

The optimal trajectory for the degree of product customization is:

20 $$\begin{aligned} \begin{aligned} {\mathrm{{G}}^{n*}}&= \left[ {{\mathrm{{G}}_0} - \mathrm{{G}}_\infty ^n - \frac{\varepsilon }{{\theta - \delta }}\left( {{\mathrm{{K}}_0} - \frac{1}{\delta }\left( {\frac{{{\mathrm{{\Delta }}_2}[{\beta _K}\left( {\rho + \theta } \right) + {\beta _G}\varepsilon ]{\lambda _D}^2}}{{{\gamma _D}\left( {\rho + \delta } \right) \left( {\rho + \theta } \right) }}} \right) } \right) } \right] {e^{ - \theta t}} \\&+\mathrm{{G}}_\infty ^n +\frac{\varepsilon }{{\theta - \delta }}\left( {{\mathrm{{K}}_0} - \frac{1}{\delta }\left( {\frac{{{\mathrm{{\Delta }}_2}[{\beta _K}\left( {\rho + \theta } \right) + {\beta _G}\varepsilon ]{\lambda _D}^2}}{{{\gamma _D}\left( {\rho + \delta } \right) \left( {\rho + \theta } \right) }}} \right) } \right) {e^{ - \delta t}}\; \end{aligned} \end{aligned}$$

where $$\mathrm{{G}}_\infty ^n = \frac{1}{\theta }\left[ {\frac{{{\mathrm{{\Delta }}_1}{\mathrm{{\beta }}_\mathrm{{G}}}{\lambda _M}^2}}{{\left( {\rho + \theta } \right) {\gamma _M}}} + \frac{{{\mathrm{{\Delta }}_3}{\beta _G}{\lambda _I}^2}}{{{\gamma _I}\left( {\rho + \theta } \right) }} + \frac{\varepsilon }{\delta }\left( {\frac{{{\mathrm{{\Delta }}_2}[{\beta _K}\left( {\rho + \theta } \right) + {\beta _G}\varepsilon ]{\lambda _D}^2}}{{{\gamma _D}\left( {\rho + \delta } \right) \left( {\rho + \theta } \right) }}} \right) } \right] $$.

#### Proof

First, the optimal profit function of the downstream integrator at time t is $\mathrm{{\Pi }}{{_I^n}^\mathrm{{*}}}\left( {E_I^n} \right) = {e^{ - \rho t}}\mathrm{{W}}{{_I^n}^\mathrm{{*}}}\left( {{K^n},{G^n}} \right)$.According to the optimal control theory, for any ${K^n} \ge 0$,${G^n} \ge 0$,$\mathrm{{W}}{{_I^n}^\mathrm{{*}}}\left( {{K^n},{G^n}} \right)$ satisfies Hamilton-Jacobi-Bellman (HJB) equation (Xu and Tan, 2019):

21 $$\begin{aligned} \begin{aligned}& \rho {\mathrm{W}}_I^n\left( {{K^n},{G^n}} \right) = \mathop {\mathrm{{max}}}\limits _{{E_I},p} \left[ {\left( {{{{\omega }}_1} - c} \right) \left( {{{\alpha }} - {{{\beta }}_\mathrm{{P}}}\mathrm{{p}}} \right) ({{{\beta }}_\mathrm{{K}}}K + {{{\beta }}_G}G} \right) - \frac{1}{2}{\gamma _I}{\left( {E_I^n} \right) ^2}\\&+ \frac{{\partial W_I^n}}{{\partial K}}\left( {{\lambda _D}\mathrm{{E}}_D^n - \delta {K^n}} \right) + \frac{{\partial W_I^n}}{{\partial G}}\left( {{\lambda _M}E_M^n + {\lambda _I}E_I^n + \varepsilon {K^n} - \theta {G^n}} \right) ]\; \end{aligned} \end{aligned}$$

Taking the first-order partial derivative in (21) with respect to ${E_I}$,p respectively and equating to zero, we can obtain:

22 $$\begin{aligned} p= & {} \frac{{{{\alpha }} + {{{\beta }}_\mathrm{{P}}}{{{\omega }}_2}}}{{2{{{\beta }}_\mathrm{{P}}}}}\; \end{aligned}$$

23 $$\begin{aligned} E_I^n= & {} \frac{{\frac{{\partial W_I^n}}{{\partial G}}{\lambda _I}}}{{{\gamma _I}}}\; \end{aligned}$$

As above, the optimal profit function of the designer at time t is $\mathrm{{\Pi }}{{_D^n}^\mathrm{{*}}}\left( {E_D^n} \right) = {e^{ - \rho t}}\mathrm{{W}}{{_D^n}^\mathrm{{*}}}\left( {{K^n},{G^n}} \right)$.According to the optimal control theory, for any ${K^n} \ge 0$,${G^n} \ge 0$,$\mathrm{{W}}{{_D^n}^\mathrm{{*}}}\left( {{K^n},{G^n}} \right)$ satisfies the HJB equation:

24 $$\begin{aligned} \begin{aligned}&\rho {\mathrm{W}}_D^n\left( {{K^n},{G^n}} \right) = \mathop {\mathrm{{max}}}\limits _{{E_D},{{{\omega }}_2}} \left[ {\left( {{{{\omega }}_1} - {{{\omega }}_2}} \right) \left( {{{\alpha }} - {{{\beta }}_\mathrm{{P}}}\mathrm{{p}}} \right) ({{{\beta }}_\mathrm{{K}}}K + {{{\beta }}_G}G} \right) - \frac{1}{2}{\gamma _D}{\left( {E_D^c} \right) ^2}\\&+ \frac{{\partial W_D^n}}{{\partial K}}\left( {{\lambda _D}\mathrm{{E}}_D^n - \delta {K^n}} \right) + \frac{{\partial W_D^n}}{{\partial G}}\left( {{\lambda _M}E_M^n + {\lambda _I}E_I^n + \varepsilon {K^n} - \theta {G^n}} \right) ]\; \end{aligned} \end{aligned}$$

Taking the first-order partial derivative in (24) with respect to ${E_D}$,${\mathrm{{\omega }}_2}$ respectively and equating to zero, we can obtain:

25 $$\begin{aligned} {{{\omega }}_2}= & {} \frac{{{{\alpha }} + {{{\beta }}_\mathrm{{P}}}{{{\omega }}_1}}}{{2{{{\beta }}_\mathrm{{P}}}}}\; \end{aligned}$$

26 $$\begin{aligned} E_D^n= & {} \frac{{\frac{{\partial W_D^n}}{{\partial K}}{\lambda _D}}}{{{\gamma _D}}}\; \end{aligned}$$

As above, the optimal profit function of the designer at time t is $\mathrm{{\Pi }}{{_M^n}^\mathrm{{*}}}\left( {E_M^n} \right) = {e^{ - \rho t}}\mathrm{{W}}{{_M^n}^\mathrm{{*}}}\left( {{K^n},{G^n}} \right)$.According to the optimal control theory, for any ${K^n} \ge 0$,${G^n} \ge 0$,$\mathrm{{W}}{{_M^n}^\mathrm{{*}}}\left( {{K^n},{G^n}} \right)$ satisfies the HJB equation:

27 $$\begin{aligned} \begin{aligned}&\rho {\mathrm{W}}_M^n\left( {{K^n},{G^n}} \right) = \mathop {\mathrm{{max}}}\limits _{{{{\omega }}_1}} \left[ {\left( {{{{\omega }}_1} - {{{\omega }}_2}} \right) \left( {{{\alpha }} - {{{\beta }}_\mathrm{{P}}}\mathrm{{p}}} \right) ({{{\beta }}_\mathrm{{K}}}K + {{{\beta }}_G}G)} \right. - \frac{1}{2}{\gamma _M}{\left( {E_M^n} \right) ^2}\\&+ \frac{{\partial W_M^n}}{{\partial K}}\left( {{\lambda _D}\mathrm{{E}}_D^n - \delta {K^n}} \right) + \frac{{\partial W_M^n}}{{\partial G}}\left( {{\lambda _M}E_M^n + {\lambda _I}E_I^n + \varepsilon {K^n} - \theta {G^n}} \right) ]\; \end{aligned} \end{aligned}$$

Taking the first-order partial derivative in (27) with respect to ${E_M}$,${\mathrm{{\omega }}_1}$ respectively and equating to zero, we can obtain:

28 $$\begin{aligned} E_M^n= & {} \frac{{\frac{{\partial W_M^n}}{{\partial G}}{\lambda _M}}}{{{\gamma _M}}}\; \end{aligned}$$

29 $$\begin{aligned} {{{\omega }}_1}^* = \frac{{{{\alpha }} + {{{\beta }}_\mathrm{{P}}}\mathrm{{c}}}}{{2{{{\beta }}_\mathrm{{P}}}}}\; \end{aligned}$$

Substituting (30) into (25) and (22), we obtain the optimal pricing of designers and integrators as follows:

30 $$\begin{aligned}{} & {} {{{\omega }}_2}^* = \frac{{3{{\alpha }} + {{{\beta }}_\mathrm{{P}}}\mathrm{{c}}}}{{4{{{\beta }}_\mathrm{{P}}}}}\; \end{aligned}$$

31 $$\begin{aligned}{} & {} {{{p}}^*} = \frac{{7{{\alpha }} + {{{\beta }}_\mathrm{{P}}}\mathrm{{c}}}}{{8{{{\beta }}_\mathrm{{P}}}}}\; \end{aligned}$$

By substituting Eqs. (23), (26), (28) and (29), (30), (31) into Eqs. (21), (24), (27) and sorting them out, it can be found that the solutions of the three HJB equations are functions of K and G respectively. So it can be assumed that the linear analytical expressions of the optimal value functions $W_M^n$,$W_D^n$ and $W_I^n$ are respectively as follows:

32 $$\begin{aligned}{} & {} \mathrm{{W}}_M^n = {\alpha _1}K + {\alpha _2}G + {\alpha _3}\; \end{aligned}$$

33 $$\begin{aligned}{} & {} \mathrm{{W}}_M^n = {\alpha _4}K + {\alpha _5}G + {\alpha _6}\; \end{aligned}$$

34 $$\begin{aligned}{} & {} \mathrm{{W}}_M^n = {\alpha _7}K + {\alpha _8}G + {\alpha _9}\; \end{aligned}$$

where $${\alpha _1} = \frac{{\partial W_M^n}}{{\partial K}}$$,$${\alpha _2} = \frac{{\partial W_M^n}}{{\partial G}}$$,$${\alpha _4} = \frac{{\partial W_D^n}}{{\partial K}}$$,$${\alpha _5} = \frac{{\partial W_D^n}}{{\partial G}}$$,$${\alpha _7} = \frac{{\partial W_I^n}}{{\partial K}}$$,$${\alpha _8} = \frac{{\partial W_I^n}}{{\partial G}}$$.

We obtain the following formula by mathematical induction method:

35 $$\begin{aligned} {\alpha _1}= & {} \frac{{{\mathrm{{\Delta }}_1}[{{{\beta }}_\mathrm{{k}}}\left( {\rho + \theta } \right) + {{{\beta }}_\mathrm{{G}}}\varepsilon ]}}{{\left( {\rho + \delta } \right) \left( {\rho + \theta } \right) }}\; \end{aligned}$$

36 $$\begin{aligned} {\alpha _2}= & {} \frac{{{{{\Delta }}_1}{{{\beta }}_\mathrm{{G}}}}}{{\left( {\rho + \theta } \right) }}\; \end{aligned}$$

37 $$\begin{aligned} {\alpha _3}= & {} \frac{1}{\rho }\left[ {\frac{{{\mathrm{{\Delta }}_1}^2{{{\beta }}_\mathrm{{G}}}^2{\lambda _M}^2}}{{2{\gamma _M}{{\left( {\rho + \theta } \right) }^2}}} + \frac{{{\mathrm{{\Delta }}_1}{\mathrm{{\Delta }}_2}{{[{{{\beta }}_\mathrm{{k}}}\left( {\rho + \theta } \right) + {{{\beta }}_\mathrm{{G}}}\varepsilon ]}^2}{\lambda _D}^2}}{{{\gamma _D}{{\left( {\rho + \delta } \right) }^2}{{\left( {\rho + \theta } \right) }^2}}} + \frac{{{\mathrm{{\Delta }}_1}{\mathrm{{\Delta }}_3}{{{\beta }}_\mathrm{{G}}}^2{\lambda _I}^2}}{{{\gamma _I}{{\left( {\rho + \theta } \right) }^2}}}} \right] \; \end{aligned}$$

38 $$\begin{aligned} {\alpha _4}= & {} \frac{{{\mathrm{{\Delta }}_2}\left[ {{{{\beta }}_\mathrm{{k}}}\left( {\rho + \theta } \right) + {{{\beta }}_{\mathrm{{G}}}}}\varepsilon  \right] }}{{\left( {\rho + \delta } \right) \left( {\rho + \theta } \right) }}\; \end{aligned}$$

39 $$\begin{aligned} {\alpha _5}= & {} \frac{{{\mathrm{{\Delta }}_2}{{{\beta }}_\mathrm{{G}}}}}{{\left( {\rho + \theta } \right) }}\; \end{aligned}$$

40 $$\begin{aligned} {\alpha _6}= & {} \frac{1}{\rho }\left[ {\frac{{{\mathrm{{\Delta }}_2}{\mathrm{{\Delta }}_1}{{{\beta }}_\mathrm{{G}}}^2{\lambda _M}^2}}{{{\gamma _M}{{\left( {\rho + \theta } \right) }^2}}} + \frac{{{\mathrm{{\Delta }}_2}^2{{[{{{\beta }}_\mathrm{{k}}}\left( {\rho + \theta } \right) + {{{\beta }}_\mathrm{{G}}}\varepsilon ]}^2}{\lambda _D}^2}}{{2{\gamma _D}{{\left( {\rho + \delta } \right) }^2}{{\left( {\rho + \theta } \right) }^2}}} + \frac{{{\mathrm{{\Delta }}_2}{\mathrm{{\Delta }}_3}{{{\beta }}_\mathrm{{G}}}^2{\lambda _I}^2}}{{{\gamma _I}{{\left( {\rho + \theta } \right) }^2}}}} \right] \; \end{aligned}$$

41 $$\begin{aligned} {\alpha _7}= & {} \frac{{{\mathrm{{\Delta }}_3}\left[ {{{{\beta }}_\mathrm{{k}}}\left( {\rho + \theta } \right) + {{{\beta }}_{\mathrm{{G}}}}}\varepsilon \right] }}{{\left( {\rho + \delta } \right) \left( {\rho + \theta } \right) }}\; \end{aligned}$$

42 $$\begin{aligned} {\alpha _8}= & {} \frac{{{\mathrm{{\Delta }}_3}{{{\beta }}_\mathrm{{G}}}}}{{\left( {\rho + \theta } \right) }}\; \end{aligned}$$

43 $$\begin{aligned} {\alpha _9}= & {} \frac{1}{\rho }\left[ {\frac{{{\mathrm{{\Delta }}_3}{\mathrm{{\Delta }}_1}{{{\beta }}_\mathrm{{G}}}^2{\lambda _M}^2}}{{{\gamma _M}{{\left( {\rho + \theta } \right) }^2}}} + \frac{{{\mathrm{{\Delta }}_2}{\mathrm{{\Delta }}_3}{{[{{{\beta }}_\mathrm{{k}}}\left( {\rho + \theta } \right) + {{{\beta }}_\mathrm{{G}}}\varepsilon ]}^2}{\lambda _D}^2}}{{{\gamma _D}{{\left( {\rho + \delta } \right) }^2}{{\left( {\rho + \theta } \right) }^2}}} + \frac{{{\mathrm{{\Delta }}_3}^2{{{\beta }}_\mathrm{{G}}}^2{\lambda _I}^2}}{{2{\gamma _I}{{\left( {\rho + \theta } \right) }^2}}}} \right] \; \end{aligned}$$

After substitution and collation, we can get the optimal decision ${E_{M}^{n}}^*$,${E_{D}^{n}}^*$,${E_{I}^{n}}^*$,the optimal profit ${\mathrm{{W}}{_M^n}}^*$,${\mathrm{{W}}{_D^n}}^*$,${\mathrm{{W}}{_I^n}}^*$ of each member of the supply chain, and the optimal innovation level ${\mathrm{{K}}^{n\mathrm{{*}}}}$ and domestic substitution level ${\mathrm{{G}}^{n\mathrm{{*}}}}$.

### Decentralized Decision-making with Cost-sharing Contract (Case c)

In the cost-sharing mode, in order to encourage integrators to participate in collaborative innovation, the designers will bear a certain percentage of compatibility costs. Assuming that the cost-sharing coefficient is $\mathrm{{\sigma \;}}\left( {0 \le \mathrm{{\sigma }} \le 1} \right)$,the objective functions of the three firms are:

44 $$\begin{aligned} \mathrm{{\Pi }}_M^c= & {} \mathop \smallint \nolimits _0^\infty {e^{ - \rho t}}\left[ {\left( {{{{\omega }}_1} - c} \right) \left( {{{\alpha }} - {{{\beta }}_\mathrm{{P}}}\mathrm{{p}}} \right) \left( {{{{\beta }}_\mathrm{{K}}}K + {{{\beta }}_G}G} \right) - \frac{1}{2}{\gamma _M}{{\left( {E_M^c} \right) }^2}} \right] dt\; \end{aligned}$$

45 $$\begin{aligned} \mathrm{{\Pi }}_D^c= & {} \mathop \smallint \nolimits _0^\infty {e^{ - \rho t}}\left[ {\left( {{{{\omega }}_2} - {{{\omega }}_1}} \right) \left( {{{\alpha }} - {{{\beta }}_\mathrm{{P}}}\mathrm{{p}}} \right) \left( {{{{\beta }}_\mathrm{{K}}}K + {{{\beta }}_G}G} \right) - \frac{1}{2}{\gamma _D}{{\left( {E_D^c} \right) }^2} - \frac{{{\sigma }}}{2}{\gamma _I}{{\left( {E_I^c} \right) }^2}} \right] dt\; \end{aligned}$$

46 $$\begin{aligned} \mathrm{{\Pi }}_I^c= & {} \mathop \smallint \nolimits _0^\infty {e^{ - \rho t}}\left[ {\left( {{{{\omega }}_2} - {{{\omega }}_1}} \right) \left( {{{\alpha }} - {{{\beta }}_\mathrm{{P}}}\mathrm{{p}}} \right) \left( {{{{\beta }}_\mathrm{{K}}}K + {{{\beta }}_G}G} \right) - \frac{{1 - {{\sigma }}}}{2}{\gamma _I}{{\left( {E_I^c} \right) }^2}} \right] dt\; \end{aligned}$$

#### Proposition 2

In the situation of decentralized decision-making without cost-sharing contracts, the equilibrium results of the differential game among manufacturers, designers and integrators are as follows:

(1) The optimal process improvement efforts of the manufacturer is:

47 $$\begin{aligned} {E_{M}^{c}}^* =\frac{{{\text { }\!\!\Delta \!\!\text { }}_{1}}{{\text { }\!\!\beta \!\!\text { }}_{\text {G}}}{{\lambda }_{M}}}{\left( \rho +\theta \right) {{\gamma }_{M}}}\; \end{aligned}$$

The optimal innovation efforts of the designer is:

48 $$\begin{aligned} {E_{D}^{c}}^* = \frac{{{\mathrm{{\Delta }}_2}\left[ {{{{\beta }}_\mathrm{{k}}}\left( {\rho + \theta } \right) + {{{\beta }}_{\mathrm{{G}}}}}\varepsilon  \right] {\lambda _D}}}{{\left( {\rho + \delta } \right) \left( {\rho + \theta } \right) {\gamma _D}}}\; \end{aligned}$$

The optimal compatibility efforts of the integrator is:

49 $$\begin{aligned} {E_{I}^{c}}^* = \frac{{{{{\beta }}_\mathrm{{G}}}\left( {2{\mathrm{{\Delta }}_3} + {{{\Delta }}_1}} \right) {\lambda _I}}}{{2{\gamma _I}\left( {\rho + \theta } \right) }}\; \end{aligned}$$

(2) The optimal price decision for each member of the supply chain and the optimal cost-sharing ratio for the designer are:

50 $$\begin{aligned} {{{{\omega }}_1}^* = \frac{{{{\alpha }} + {{{\beta }}_\mathrm{{P}}}\mathrm{{c}}}}{{2{{{\beta }}_\mathrm{{P}}}}}}\; \end{aligned}$$

The optimal innovation efforts of the designer is:

51 $$\begin{aligned} {{{{\omega }}_2}^* = \frac{{3{{\alpha }} + {{{\beta }}_\mathrm{{P}}}\mathrm{{c}}}}{{4{{{\beta }}_\mathrm{{P}}}}}}\; \end{aligned}$$

The optimal compatibility efforts of the integrator is:

52 $$\begin{aligned} {{{p}}^*}= & {} \frac{{7{{\alpha }} + {{{\beta }}_\mathrm{{P}}}\mathrm{{c}}}}{{8{{{\beta }}_\mathrm{{P}}}}}\; \end{aligned}$$

53 $$\begin{aligned} {{{\sigma }}^*}= & {} \frac{{2{\mathrm{{\Delta }}_2} - {\mathrm{{\Delta }}_3}}}{{2{\mathrm{{\Delta }}_2} + {\mathrm{{\Delta }}_3}}}\; \end{aligned}$$

(3)The optimal profits of each member in the supply chain are as follows:

54 $$\begin{aligned}{} & {} \begin{aligned} {{{W}}{_M^c}}^*&= \frac{{{\mathrm{{\Delta }}_1}[{{{\beta }}_\mathrm{{k}}}\left( {\rho + \theta } \right) + {{{\beta }}_\mathrm{{G}}}\varepsilon ]}}{{\left( {\rho + \delta } \right) \left( {\rho + \theta } \right) }}K + \frac{{{\mathrm{{\Delta }}_1}{{{\beta }}_\mathrm{{G}}}}}{{\left( {\rho + \theta } \right) }}G + \\&\frac{1}{\rho }\left[ {\frac{{{\mathrm{{\Delta }}_1}^2{{{\beta }}_\mathrm{{G}}}^2{\lambda _M}^2}}{{2{\gamma _M}{{\left( {\rho + \theta } \right) }^2}}} + \frac{{{\mathrm{{\Delta }}_1}{\mathrm{{\Delta }}_2}{{[{{{\beta }}_\mathrm{{k}}}\left( {\rho + \theta } \right) + {{{\beta }}_\mathrm{{G}}}\varepsilon ]}^2}{\lambda _D}^2}}{{{\gamma _D}{{\left( {\rho + \delta } \right) }^2}{{\left( {\rho + \theta } \right) }^2}}} + \frac{{{\mathrm{{\Delta }}_1}\left( {2{\mathrm{{\Delta }}_2} + {\mathrm{{\Delta }}_3}} \right) {{{\beta }}_\mathrm{{G}}}^2{\lambda _I}^2}}{{2{\gamma _I}{{\left( {\rho + \theta } \right) }^2}}}} \right] \; \end{aligned} \end{aligned}$$

55 $$\begin{aligned}{} & {} \begin{aligned} {{{W}}{_M^c}}^*&= \frac{{{\mathrm{{\Delta }}_1}[{{{\beta }}_\mathrm{{k}}}\left( {\rho + \theta } \right) + {{{\beta }}_\mathrm{{G}}}\varepsilon ]}}{{\left( {\rho + \delta } \right) \left( {\rho + \theta } \right) }}K + \frac{{{\mathrm{{\Delta }}_1}{{{\beta }}_\mathrm{{G}}}}}{{\left( {\rho + \theta } \right) }}G + \\&\frac{1}{\rho }\left[ {\frac{{{\mathrm{{\Delta }}_1}^2{{{\beta }}_\mathrm{{G}}}^2{\lambda _M}^2}}{{2{\gamma _M}{{\left( {\rho + \theta } \right) }^2}}} + \frac{{{\mathrm{{\Delta }}_1}{\mathrm{{\Delta }}_2}{{[{{{\beta }}_\mathrm{{k}}}\left( {\rho + \theta } \right) + {{{\beta }}_\mathrm{{G}}}\varepsilon ]}^2}{\lambda _D}^2}}{{{\gamma _D}{{\left( {\rho + \delta } \right) }^2}{{\left( {\rho + \theta } \right) }^2}}} + \frac{{{\mathrm{{\Delta }}_1}\left( {2{\mathrm{{\Delta }}_2} + {\mathrm{{\Delta }}_3}} \right) {{{\beta }}_\mathrm{{G}}}^2{\lambda _I}^2}}{{2{\gamma _I}{{\left( {\rho + \theta } \right) }^2}}}} \right] \; \end{aligned} \end{aligned}$$

56 $$\begin{aligned}{} & {} \begin{aligned} {{{W}}{_D^c}}^*&= \frac{{{\mathrm{{\Delta }}_2}\left[ {{{{\beta }}_\mathrm{{k}}}\left( {\rho + \theta } \right) + {{{\beta }}_\mathrm{{G}}}} \right] }}{{\left( {\rho + \delta } \right) \left( {\rho + \theta } \right) }}K + \frac{{{\mathrm{{\Delta }}_2}{{{\beta }}_\mathrm{{G}}}}}{{\left( {\rho + \theta } \right) }}G + \\&\frac{1}{\rho }\left[ {\frac{{{\mathrm{{\Delta }}_1}{\mathrm{{\Delta }}_2}{\lambda _M}^2}}{{{\gamma _M}{{\left( {\rho + \theta } \right) }^2}}} + \frac{{{\mathrm{{\Delta }}_2}^2{{[{{{\beta }}_\mathrm{{k}}}\left( {\rho + \theta } \right) + {{{\beta }}_\mathrm{{G}}}\varepsilon ]}^2}{\lambda _D}^2}}{{2{\gamma _D}{{\left( {\rho + \delta } \right) }^2}{{\left( {\rho + \theta } \right) }^2}}} + \frac{{{{\left( {2{\mathrm{{\Delta }}_2} + {\mathrm{{\Delta }}_3}} \right) }^2}{{{\beta }}_\mathrm{{G}}}^2{\lambda _I}^2}}{{8{\gamma _I}{{\left( {\rho + \theta } \right) }^2}}}} \right] \; \end{aligned} \end{aligned}$$

57 $$\begin{aligned}{} & {} \begin{aligned} {{{W}}{_I^c}}^*&= \frac{{{\mathrm{{\Delta }}_3}\left[ {{{{\beta }}_\mathrm{{k}}}\left( {\rho + \theta } \right) + {{{\beta }}_\mathrm{{G}}}} \right] }}{{\left( {\rho + \delta } \right) \left( {\rho + \theta } \right) }}K + \frac{{{\mathrm{{\Delta }}_1}{{{\beta }}_\mathrm{{G}}}}}{{\left( {\rho + \theta } \right) }}G + \\&\frac{1}{\rho }\left[ {\frac{{{\mathrm{{\Delta }}_3}{\mathrm{{\Delta }}_1}{\lambda _M}^2{{{\beta }}_\mathrm{{G}}}^2}}{{{\gamma _M}{{\left( {\rho + \theta } \right) }^2}}} + \frac{{{\mathrm{{\Delta }}_3}{\mathrm{{\Delta }}_2}{{[{{{\beta }}_\mathrm{{k}}}\left( {\rho + \theta } \right) + {{{\beta }}_\mathrm{{G}}}\varepsilon ]}^2}{\lambda _D}^2}}{{{\gamma _D}{{\left( {\rho + \delta } \right) }^2}{{\left( {\rho + \theta } \right) }^2}}} + \frac{{{\mathrm{{\Delta }}_3}\left( {2{\mathrm{{\Delta }}_2} + {\mathrm{{\Delta }}_3}} \right) {{{\beta }}_\mathrm{{G}}}^2{\lambda _I}^2}}{{4{\gamma _I}{{\left( {\rho + \theta } \right) }^2}}}} \right] \; \end{aligned} \end{aligned}$$

(4) The optimal trajectory of the MCU innovation level is:

58 $$\begin{aligned} {{\mathrm{{K}}^{c*}} = \left[ {{\mathrm{{K}}_0} - \mathrm{{K}}_\infty ^c} \right] {e^{ - \delta t}} + \mathrm{{K}}_\infty ^c}\; \end{aligned}$$

where $$\mathrm{{K}}_\infty ^c = \frac{1}{\delta }\left( {\frac{{{\mathrm{{\Delta }}_2}[{\beta _K}\left( {\rho + \theta } \right) + {\beta _G}\varepsilon ]{\lambda _D}^2}}{{{\gamma _D}\left( {\rho + \delta } \right) \left( {\rho + \theta } \right) }}} \right) $$.

The optimal trajectory for the degree of product customization is:

59 $$\begin{aligned} \begin{aligned} {\mathrm{{G}}^{c*}}&= \left[ {{\mathrm{{G}}_0} - \mathrm{{G}}_\infty ^c - \frac{\varepsilon }{{\theta - \delta }}\left( {{\mathrm{{K}}_0} - \frac{1}{\delta }\left( {\frac{{{\mathrm{{\Delta }}_2}[{\beta _K}\left( {\rho + \theta } \right) + {\beta _G}\varepsilon ]{\lambda _D}^2}}{{{\gamma _D}\left( {\rho + \delta } \right) \left( {\rho + \theta } \right) }}} \right) } \right) } \right] {e^{ - \theta t}} + \mathrm{{G}}_\infty ^c\\&+\frac{\varepsilon }{{\theta - \delta }}\left( {{\mathrm{{K}}_0} - \frac{1}{\delta }\left( {\frac{{{\mathrm{{\Delta }}_2}[{\beta _K}\left( {\rho + \theta } \right) + {\beta _G}\varepsilon ]{\lambda _D}^2}}{{{\gamma _D}\left( {\rho + \delta } \right) \left( {\rho + \theta } \right) }}} \right) } \right) {e^{ - \delta t}}\; \end{aligned} \end{aligned}$$

where $$\mathrm{{G}}_\infty ^c = \frac{1}{\theta }\left[ {\frac{{{\mathrm{{\Delta }}_1}{{{\beta }}_\mathrm{{G}}}{\lambda _M}^2}}{{\left( {\rho + \theta } \right) {\gamma _M}}} + \frac{{{{{\beta }}_\mathrm{{G}}}\left( {2{\mathrm{{\Delta }}_2} + {\mathrm{{\Delta }}_3}} \right) {\lambda _I}^2}}{{2{\gamma _I}\left( {\rho + \theta } \right) }} + \frac{\varepsilon }{\delta }\left( {\frac{{{\mathrm{{\Delta }}_2}[{\beta _K}\left( {\rho + \theta } \right) + {\beta _G}\varepsilon ]{\lambda _D}^2}}{{{\gamma _D}\left( {\rho + \delta } \right) \left( {\rho + \theta } \right) }}} \right) } \right] $$.

#### Proof

The proof process is similar to Proposition 1, so it will be omitted here.

### Centralized Decision-making (Case t)

In the cooperative mode, the member companies in the supply chain will determine their own optimal decisions to maximise the supply chain’s overall profit. At this point, the optimal control problem for the supply chain is:

60 $$\begin{aligned} \begin{aligned} \mathrm{{\Pi }}_T^t&= \mathop \smallint \nolimits _0^\infty {e^{ - \rho t}}[\left( {p - c} \right) \left( {{{\alpha }} - {{{\beta }}_\mathrm{{P}}}\mathrm{{p}}} \right) ({{{\beta }}_\mathrm{{K}}}K + {{{\beta }}_G}G)\\&- \frac{1}{2}{\gamma _M}{\left( {E_M^t} \right) ^2} - \frac{1}{2}{\gamma _D}{\left( {E_D^t} \right) ^2} - \frac{1}{2}{\gamma _I}{\left( {E_I^t} \right) ^2}]dt\; \end{aligned} \end{aligned}$$

#### Proposition 3

In this case, the optimal decision for each member of the supply chain is:

(1) The optimal process improvement efforts of the manufacturer is:

61 $$\begin{aligned} {E_{M}^{t}}^* =\frac{{{\text { }\!\!\Delta \!\!\text { }}_{4}}{{\text { }\!\!\beta \!\!\text { }}_{\text {G}}}{{\lambda }_{M}}}{\left( \rho +\theta \right) {{\gamma }_{M}}}\; \end{aligned}$$

The optimal innovation efforts of the designer is:

62 $$\begin{aligned} {E_{D}^{t}}^* = \frac{{{\mathrm{{\Delta }}_4}\left[ {{{{\beta }}_\mathrm{{k}}}\left( {\rho + \theta } \right) + {{{\beta }}_{\mathrm{{G}}}}}\varepsilon  \right] {\lambda _D}}}{{\left( {\rho + \delta } \right) \left( {\rho + \theta } \right) {\gamma _D}}}\; \end{aligned}$$

The optimal compatibility efforts of the integrator is:

63 $$\begin{aligned} {E_{I}^{t}}^* =\frac{{{\text { }\!\!\Delta \!\!\text { }}_{4}}{{\text { }\!\!\beta \!\!\text { }}_{\text {G}}}{{\lambda }_{M}}}{\left( \rho +\theta \right) {{\gamma }_{I}}}\; \end{aligned}$$

where ${\mathrm{{\Delta }}_4} = \left( {\mathrm{{p}} - c} \right) \left( {\mathrm{{\alpha }} - {\mathrm{{\beta }}_\mathrm{{P}}}\mathrm{{p}}} \right) = \frac{{{{\left( {\mathrm{{\alpha }} - {\mathrm{{\beta }}_\mathrm{{P}}}\mathrm{{c}}} \right) }^2}}}{{\mathrm{{4}}{\mathrm{{\beta }}_\mathrm{{P}}}}}$. (2) The optimal price decision for the designer is:

64 $$\begin{aligned} {p^*} = \frac{{\alpha + {{{\beta }}_\mathrm{{P}}}c}}{{2{{{\beta }}_\mathrm{{P}}}}}\; \end{aligned}$$

(3) In this situation, the optimal total profit of the supply chain is:

65 $$\begin{aligned} \begin{aligned} {\mathrm{{W}}{_T^t}}^*&= \frac{{{\mathrm{{\Delta }}_4}[{\beta _K}\left( {\rho + \theta } \right) + {\beta _G}\varepsilon ]}}{{\left( {\rho + \delta } \right) \left( {\rho + \theta } \right) }}{K^t} + \frac{{{\mathrm{{\Delta }}_4}{\beta _G}}}{{\rho + \theta }}{G^t} + \\&\frac{1}{\rho }\left[ {\frac{{{\mathrm{{\Delta }}_4}^2{\beta _G}^2{\lambda _M}^2}}{{2{\gamma _M}{{\left( {\rho + \theta } \right) }^2}}} + \frac{{{\mathrm{{\Delta }}_4}^2{{[{\beta _K}\left( {\rho + \theta } \right) + {\beta _G}\varepsilon ]}^2}{\lambda _D}^2}}{{2{\gamma _D}{{\left( {\rho + \delta } \right) }^2}{{\left( {\rho + \theta } \right) }^2}}} + \frac{{{\mathrm{{\Delta }}_4}^2{\beta _G}^2{\lambda _I}^2}}{{2{\gamma _I}{{\left( {\rho + \theta } \right) }^2}}}} \right] \; \end{aligned} \end{aligned}$$

(4) The optimal trajectory of the MCU co-innovation level is: The optimal trajectory of the MCU innovation level is:

66 $$\begin{aligned} {{\mathrm{{K}}^{t*}} = \left[ {{\mathrm{{K}}_0} - \mathrm{{K}}_\infty ^t} \right] {e^{ - \delta t}} + \mathrm{{K}}_\infty ^t}\; \end{aligned}$$

where $$\mathrm{{K}}_\infty ^t = \frac{1}{\delta }\left( {\frac{{{\mathrm{{\Delta }}_4}[{\beta _K}\left( {\rho + \theta } \right) + {\beta _G}\varepsilon ]{\lambda _D}^2}}{{{\gamma _D}\left( {\rho + \delta } \right) \left( {\rho + \theta } \right) }}} \right) $$.

The optimal trajectory for the degree of product customization is:

67 $$\begin{aligned} \begin{aligned} {\mathrm{{G}}^{t*}}&= \left[ {{\mathrm{{G}}_0} - \mathrm{{G}}_\infty ^t - \frac{\varepsilon }{{\theta - \delta }}\left( {{\mathrm{{K}}_0} - \frac{1}{\delta }\left( {\frac{{{\mathrm{{\Delta }}_4}[{\beta _K}\left( {\rho + \theta } \right) + {\beta _G}\varepsilon ]{\lambda _D}^2}}{{{\gamma _D}\left( {\rho + \delta } \right) \left( {\rho + \theta } \right) }}} \right) } \right) } \right] {e^{ - \theta t}} + \mathrm{{G}}_\infty ^t\\&+ \frac{\varepsilon }{{\theta - \delta }}\left( {{\mathrm{{K}}_0} - \frac{1}{\delta }\left( {\frac{{{\mathrm{{\Delta }}_4}[{\beta _K}\left( {\rho + \theta } \right) + {\beta _G}\varepsilon ]{\lambda _D}^2}}{{{\gamma _D}\left( {\rho + \delta } \right) \left( {\rho + \theta } \right) }}} \right) } \right) {e^{ - \delta t}}\; \end{aligned} \end{aligned}$$

where $$\mathrm{{G}}_\infty ^t = \frac{1}{\theta }\left[ {\frac{{{\mathrm{{\Delta }}_4}{\beta _G}{\lambda _M}^2}}{{{\gamma _M}\left( {\rho + \theta } \right) }} + \frac{{{\mathrm{{\Delta }}_4}{\beta _G}{\lambda _I}^2}}{{{\gamma _I}\left( {\rho + \theta } \right) }} + \frac{\varepsilon }{\delta }\left( {\frac{{{\mathrm{{\Delta }}_4}[{\beta _K}\left( {\rho + \theta } \right) + {\beta _G}\varepsilon ]{\lambda _D}^2}}{{{\gamma _D}\left( {\rho + \delta } \right) \left( {\rho + \theta } \right) }}} \right) } \right] $$.

#### Proof

The proof process is similar to Proposition 1, so it will be omitted here.

## Comparative Analysis and Supply Chain Coordination

### Comparison and Analysis

First, a sensitivity analysis was performed. By finding the first derivatives of the integrator’s process improvement effort, the designer’s innovation effort, the integrator’s compatibility effort and the levels of innovation and domestic substitution with respect to relevant parameters, we can obtain Table 2.

**Table 2:** Sensitivity analysis

Variables	$\text { }\!\!\alpha \!\!\text { }$	$\mathrm{{\rho }}$	$\mathrm{{\delta }}$	$\mathrm{{\theta }}$	${\beta _P}$	${\beta _K}$	${\beta _G}$	${\gamma _M}$	${\gamma _D}$	${\gamma _I}$	${\lambda _M}$	${\lambda _D}$	${\lambda _I}$	$\mathrm{{\varepsilon }}$
${E_I}^*$	$\uparrow$	$\downarrow$	–	$\downarrow$	$\downarrow$	–	$\uparrow$	–	–	$\downarrow$	–	–	$\uparrow$	–
${E_D}^*$	$\uparrow$	$\downarrow$	$\downarrow$	$\downarrow$	$\downarrow$	$\uparrow$	$\uparrow$	–	$\downarrow$	–	–	$\uparrow$	–	$\uparrow$
${E_M}^*$	$\uparrow$	$\downarrow$	–	$\downarrow$	$\downarrow$	–	$\uparrow$	$\downarrow$	–	–	$\uparrow$	–	–	–
${K_\infty }^*$	$\uparrow$	$\downarrow$	$\downarrow$	$\downarrow$	$\downarrow$	$\uparrow$	$\uparrow$	–	$\downarrow$	–	–	$\uparrow$	–	$\uparrow$
${G_\infty }^*$	$\uparrow$	$\downarrow$	$\downarrow$	$\downarrow$	$\downarrow$	$\uparrow$	$\uparrow$	$\downarrow$	$\downarrow$	$\downarrow$	$\uparrow$	$\uparrow$	$\uparrow$	$\uparrow$

"$\uparrow$" means positive correlation; "$\downarrow$" means negative correlation; "–" means irrelevant

#### Corollary 1

As shown in Table 2, ${{E}_{D}}^{*}$ and ${{K}_{\infty }}^{*}$ are positively correlated with $\text { }\!\!\alpha \!\!\text { }$ , ${{\beta }_{K}}$ , ${{\beta }_{G}}$ , ${{\lambda }_{D}}$ and $\varepsilon$, negatively correlated with $\rho$, $\delta$, $\theta$, ${{\beta }_{P}}$, ${{\gamma }_{D}}$. ${{E}_{I}}^{*}$ is positively correlated with $\text { }\!\!\alpha \!\!\text { }$ , ${{\beta }_{G}}$ and ${{\lambda }_{I}}$, negatively correlated with $\rho$,$\text { }\!\!\theta \!\!\text { }$, ${{\beta }_{P}}$ and ${{\gamma }_{I}}$. ${{E}_{M}}^{*}$ is positely correlated with $\text { }\!\!\alpha \!\!\text { }$ , ${{\beta }_{G}}$ and ${{\lambda }_{M}}$, negatively correlated with $\rho$, $\text { }\!\!\theta \!\!\text { }$, ${{\beta }_{P}}$ and ${{\gamma }_{M}}$. ${{G}_{\infty }}^{*}$ is positively correlated with $\text { }\!\!\alpha \!\!\text { }$ , ${{\beta }_{K}}$, ${{\beta }_{G}}$, ${{\lambda }_{M}}$, ${{\lambda }_{D}}$, ${{\lambda }_{I}}$ and $\text { }\!\!\varepsilon \!\!\text { }$, negatively correlated with $\text { }\!\!\rho \!\!\text { }$, $\text { }\!\!\theta \!\!\text { }$, ${{\beta }_{P}}$ and ${{\gamma }_{M}}$, ${{\gamma }_{D}}$, ${{\gamma }_{I}}$.

The proof is presented in “Appendix 1”.

As can be seen from Corollary 1, the stable value of the innovation level and the trend of the relevant parameters are entirely consistent with the designer’s innovation effort, which implies that the level of designer effort is a determining factor in the level of innovation. As the level of innovation influences market demand and the level of domestic substitution increases, so makes the designer’s effort to innovate and the level of product innovation. Furthermore, the lower the cost of innovation, the slower the decay in the level of innovation and the level of domestic substitution, and the more willing designers are to invest in the innovation effort.

Corollary 1 also illustrates that manufacturers’ process improvement efforts and integrators’ compatibility efforts essentially follow the same trend, both increasing with the size of the market, the factors influencing the level of domestic substitution to market demand, and the factors influencing process improvement efforts and compatibility efforts to the level of domestic substitution. This means that the more sensitive market demand is to the level of domestic substitution, the more manufacturers and integrators will increase their collaborative innovation efforts, i.e. process improvement and compatibility efforts. As market demand becomes more sensitive to price, cost and discount factors and the rate of decline in the level of domestic substitution increases, manufacturers’ process improvement and integrators’ compatibility efforts decrease. This means that the more manufacturers and integrators pay for co-innovation, the more resistance they have to participate in co-innovation. Therefore, the less process improvement effort and integration effort they put in. And as the level of domestic substitution decreases slower, manufacturers and integrators will increase their process improvement and compatibility efforts and thus better engage in co-innovation.

Based on the trend of the G variable on the variables of interest, it can be seen that the level of domestic substitution decreases as the discount factor, the rate of decline in the level of innovation, and the rate of decline in the level of domestic substitution increase. This means that as market competition becomes more intense, the decay rate of innovation level and domestic substitution level will accelerate, which will negatively impact the domestic substitution level of ICs. At this time, MCU supply chain member companies should increase their collaborative innovation investment to improve their competitiveness.

Next, by comparing and analyzing the optimal strategy and profit of supply chain members in the three cases, as well as the innovation level of the supply chain with the domestic substitution level, the following relevant inferences can be obtained:

#### Corollary 2

$$\mathrm{{E}}{{_D^n}^*} = \mathrm{{E}}{{_D^c}^*} < \mathrm{{E}}{{_D^t}^*}$$, $${\mathrm{{K}}^n}^* = {\mathrm{{K}}^c}^* < {\mathrm{{K}}^t}^*$$.

The proof is presented in “Appendix 2”.

Corollary 2 illustrates that in the cooperative mode, the designer’s innovation effort and the supply chain’s innovation level reach the maximum, the cost-sharing mode is the next, and the non-cooperative mode is the smallest. Moreover, the cost-sharing contract keeps the designer’s innovation efforts the same. This suggests that the level of innovation in the supply chain is only related to the innovation input of the designer and is not affected by other factors. Additionally, the fact that the designer bears part of the compatibility cost of the integrator does not affect its innovation input.

#### Corollary 3

$$\mathrm{{E}}{{_M^n}^*} = \mathrm{{E}}{{_M^c}^*}< \mathrm{{E}}{{_M^t}^*},\mathrm{{E}}{{_I^n}^*}< \mathrm{{E}}{{_I^c}^*} < \mathrm{{E}}{{_I^t}^*}$$,$${\mathrm{{G}}^n}^*< {\mathrm{{G}}^c}^* < {\mathrm{{G}}^t}^*$$.

The proof is presented in “Appendix 3”.

Corollary 3 indicates that in the case of cooperative mode, the compatibility efforts of integrators, the process improvement efforts of manufacturers and the feasibility of MCU domestic substitution reach the maximum. When designers bear the compatibility costs of integrators, the compatibility efforts of integrators and the level of domestic substitution increase, which indicates that the cost-sharing contract dominated by designers can promote integrators to increase compatibility investment, which is conducive to the formation of the ecosystem of domestic MCU.

#### Corollary 4

$$\mathrm{{W}}{{_M^n}^*} < \mathrm{{W}}{{_M^c}^*}$$,$$\mathrm{{W}}{{_D^n}^*} < \mathrm{{W}}{{_D^c}^*}$$,$$\mathrm{{W}}{{_I^n}^*} < \mathrm{{W}}{{_I^c}^*}$$;$$\mathrm{{W}}{{_M^n}^*} + \mathrm{{W}}{{_D^n}^*} + \mathrm{{W}}{{_I^n}^*}< \mathrm{{W}}{{_M^c}^*} + \mathrm{{W}}{{_D^c}^*} + \mathrm{{W}}{{_I^c}^*} < \mathrm{{W}}{{_T^t}^*}$$.

The proof is presented in “Appendix 4”.

Corollary 4 shows that in both scenarios of decentralized decision-making, the designer-led cost-sharing contract does not diminish the profits of the designer and manufacturer while increasing the profits of the downstream integrator but instead promotes the increased profits of the designer and upstream manufacturer. This may be due to the active participation of the integrator increasing the degree of local substitution of MCU, which promotes the growth of market demand and thus boosts profits. In addition, the total profit of the supply chain is maximized in the case of centralized decision-making.

### Supply Chain Coordination

The conclusion drawn from Sect. 5.1 shows that the optimal decision and profit of each supply chain member are optimized under centralized decision-making. Although cost-sharing contracts can improve integrator compatibility efforts and domestic substitution, each supply chain member’s optimal strategies and supply chain performance under decentralized decision-making are still much smaller than under centralized decision-making. However, in reality, the scenario of centralized decision-making where all supply chain members have the overall profit maximization as their goal is more difficult to achieve (Cheng et al., 2020). Therefore, in this section, we improve the cost-sharing contract mentioned in Sect. 4.2 by utilizing a two-part pricing contract, which hopefully will lead to a change in supply chain performance.

The specific *two-part pricing + cost-sharing* contract are as follows: For the integrator’s compatible efforts under cost-sharing to reach $E_{I}^{t}$, the level of centralized decision making, designers will bear some of the cost of the integrator’s compatibility efforts. And the two-part pricing contract is in the form of $T~={{\omega }_{2}}D+{{F}^{tpc}}$,where$\;{\omega _2}$ is the unit wholesale price and ${{F}^{tpc}}$ is the fixed cost. On the one hand, ${{F}^{tpc}}$ is a portion of the fixed fee compensated to the designer by the integrator to make up for the loss of profit due to the designer’s assumption of compatibility costs. On the other hand, setting a fixed fee can act as a profit-sharing mechanism so that the profits of the supply chain members after coordination are greater than those in the cost-sharing scenario.

#### Proposition 4

When the cost sharing ratio satisfies $\mathrm{{\sigma }}{\mathrm{{'}}^*} = \frac{{{\mathrm{{\Delta }}_4} - {\mathrm{{\Delta }}_3}}}{{{\mathrm{{\Delta }}_4}}}$ and the fixed cost is controlled within a reasonable range

$$\begin{aligned} {F^{tpc}} \in \left[ \begin{array}{l} \frac{{11{\lambda _I}^2{{\left( {\mathrm{{\alpha }} - {\mathrm{{\beta }}_\mathrm{{P}}}\mathrm{{c}}} \right) }^4}{\mathrm{{\beta }}_\mathrm{{G}}}^2}}{{2048\rho {{(\rho + \theta )}^2}{\gamma _I}{\mathrm{{\beta }}_\mathrm{{P}}}^2}} - \frac{{{{(\mathrm{{\alpha }} - {\mathrm{{\beta }}_\mathrm{{P}}}\mathrm{{c}})}^2}}}{{64{\mathrm{{\beta }}_\mathrm{{P}}}{\mathrm{{\beta }}_\mathrm{{G}}}}}({G^{T{P^*}}} - {G^{{c^*}}}),\\ \frac{{{{(\mathrm{{\alpha }} - {\mathrm{{\beta }}_\mathrm{{P}}}\mathrm{{c}})}^2}}}{{\mathrm{{16}}{\mathrm{{\beta }}_\mathrm{{P}}}{\mathrm{{\beta }}_\mathrm{{G}}}}}({G^{T{P^*}}} - {G^{{c^*}}}) + \frac{{5{{\left( {\mathrm{{\alpha }} - {\mathrm{{\beta }}_\mathrm{{P}}}\mathrm{{c}}} \right) }^4}{\mathrm{{\beta }}_\mathrm{{G}}}^2}}{{16384\rho {{(\rho + \theta )}^2}{\gamma _I}{\mathrm{{\beta }}_\mathrm{{P}}}^2}} \end{array} \right] \end{aligned}$$

the *cost-sharing + two-part pricing* contract can achieve supply chain coordination and be accepted by supply chain members.

#### Proof

The objective function for each member of the supply chain is:

68 $$\begin{aligned}{} & {} \begin{aligned} \mathrm{{\Pi }}_M^{TP} = \mathop \smallint \nolimits _0^\infty {e^{ - \rho t}}\left[ {\left( {{{{\omega }}_1} - c} \right) \left( {{{\alpha }} - {{{\beta }}_\mathrm{{P}}}\mathrm{{p}}} \right) \left( {{{{\beta }}_\mathrm{{K}}}K + {{{\beta }}_G}G} \right) - \frac{1}{2}{\gamma _M}{{\left( {E_M^c} \right) }^2}} \right] dt \end{aligned} \end{aligned}$$

69 $$\begin{aligned}{} & {} \begin{aligned} \mathrm{{\Pi }}_D^{TP} = \mathop \smallint \nolimits _0^\infty {e^{ - \rho t}}\left[ {\left( {{{{\omega }}_2} - {{{\omega }}_1}} \right) \left( {{{\alpha }} - {{{\beta }}_\mathrm{{P}}}\mathrm{{p}}} \right) \left( {{{{\beta }}_\mathrm{{K}}}K + {{{\beta }}_G}G} \right) - \frac{1}{2}{\gamma _D}{{\left( {E_D^c} \right) }^2} - \frac{{{{\sigma '}}}}{2}{\gamma _I}{{\left( {E_I^c} \right) }^2}} \right] dt + {F^{tpc}} \end{aligned} \end{aligned}$$

70 $$\begin{aligned}{} & {} \begin{aligned} \mathrm{{\Pi }}_I^{TP} = \mathop \smallint \nolimits _0^\infty {e^{ - \rho t}}\left[ {\left( {p - {{{\omega }}_2}} \right) \left( {{{\alpha }} - {{{\beta }}_\mathrm{{P}}}\mathrm{{p}}} \right) \left( {{{{\beta }}_\mathrm{{K}}}K + {{{\beta }}_G}G} \right) - \frac{{1 - {{\sigma '}}}}{2}{\gamma _I}{{\left( {E_I^c} \right) }^2}} \right] dt - {F^{tpc}} \end{aligned} \end{aligned}$$

For the *cost-sharing + two-part pricing* contract to improve supply chain performance and for all supply chain members to accept the contract, the following conditions should be met:

71 $$\begin{aligned} \begin{aligned} \left\{ \begin{array}{l} E_I^{tp{c^*}} = E_I^{{t^*}}\\ \mathrm{{\Pi }}_D^{tp{c^*}} \ge \mathrm{{\Pi }}_D^{{t^*}}\\ \mathrm{{\Pi }}_I^{tp{c^*}} \ge \mathrm{{\Pi }}_I^{{t^*}} \end{array} \right. \end{aligned} \end{aligned}$$

The solution process is similar to that in Sect. 4.1 and will not be repeated here. By the inverse solution method, we can obtain:

72 $$\begin{aligned}{} & {} \begin{aligned} {{\sigma }}{{{'}}^*} = \frac{{{\mathrm{{\Delta }}_4} - {\mathrm{{\Delta }}_3}}}{{{\mathrm{{\Delta }}_4}}} \end{aligned} \end{aligned}$$

73 $$\begin{aligned}{} & {} \begin{aligned} {F^{tpc}} \ge F_1^{tpc} = \frac{{11{\lambda _I}^2{{\left( {{{\alpha }} - {{{\beta }}_\mathrm{{P}}}\mathrm{{c}}} \right) }^4}{{{\beta }}_\mathrm{{G}}}^2}}{{2048\rho {{(\rho + \theta )}^2}{\gamma _I}{{{\beta }}_\mathrm{{P}}}^2}} - \frac{{{{({{\alpha }} - {{{\beta }}_\mathrm{{P}}}\mathrm{{c}})}^2}}}{{64{{{\beta }}_\mathrm{{P}}}{{{\beta }}_\mathrm{{G}}}}}({G^{T{P^*}}} - {G^{{c^*}}}) \end{aligned} \end{aligned}$$

74 $$\begin{aligned}{} & {} \begin{aligned} {F^{tpc}} \le F_2^{tpc} = \frac{{{{({{\alpha }} - {{{\beta }}_\mathrm{{P}}}\mathrm{{c}})}^2}}}{{\mathrm{{16}}{{{\beta }}_\mathrm{{P}}}{{{\beta }}_\mathrm{{G}}}}}({G^{T{P^*}}} - {G^{{c^*}}}) + \frac{{5{{\left( {{{\alpha }} - {{{\beta }}_\mathrm{{P}}}\mathrm{{c}}} \right) }^4}{{{\beta }}_\mathrm{{G}}}^2}}{{16384\rho {{(\rho + \theta )}^2}{\gamma _I}{{{\beta }}_\mathrm{{P}}}^2}} \end{aligned} \end{aligned}$$

## Numerical Simulation

To further validate the previous findings, this section analyses and compares the optimal strategies and profits of each firm under three different decision scenarios with the help of MATLAB and gives managerial implications. We divided the parameters into five categories based on research of Guan et al. (2020), demand parameters, cost parameters, level parameters, dynamic parameters and initial values. The relevant parameter values are shown in Table 3.

**Table 3:** Model parameters

Type of parameters	Values			
Demand parameters	$\alpha =100$	${{\beta }_{K}}=0.5$	${{\beta }_{G}}=0.5$	${{\beta }_{P}}=0.8$
Cost parameters	${{\gamma }_{M}}=3$	${{\gamma }_{D}}=4$	${{\gamma }_{I}}=3$	$c=1$
Level parameters	${{\lambda }_{M}}=0.3$	${{\lambda }_{D}}=0.4$	${{\lambda }_{I}}=0.3$	$\varepsilon =0.2$
Dynamic parameters	$\rho =0.1$	$\delta =0.2$	$\theta =0.4$	
Initial values	${{K}_{0}}=0$	${{G}_{0}}=10$		

The demand parameters, innovation and domestic substitution level parameters and manufacturing cost c were obtained from a consultation and market survey conducted by CX, a Chinese high-tech company engaged in the design and sale of integrated circuits, with whom we have a project collaboration. According to CX, wholesale prices for MCUs from mainland Chinese manufacturers range from $30-70. Combined with the optimal pricing conclusions in Propositions 1, 2 and 3, it is clear that setting manufacturing costs $$c=1$$ is realistic.

The other parameters are taken from previous studies on supply chain cooperation and technological innovation in China (Liu et al., 2020; Wang and Wang, 2021; Lu et al., 2020; Ma et al., 2019).

The equilibrium results of the game in the three decision scenarios are shown in Table 4. According to Table 4, we can obtain the following conclusions.

**Table 4:** Comparison of equilibrium results

Equilibrium results	Non-cooperative mode	Cost-sharing mode	Cooperative mode
${E_M}\left( t \right)$	76.88	76.88	307.52
${E_D}\left( t \right)$	89.69	89.69	717.55
${E_I}\left( t \right)$	19.22	57.66	307.52
$\mathrm{{K}}\left( \mathrm{{t}} \right)$	32.43	32.43	259.42
$\mathrm{{G}}\left( \mathrm{{t}} \right)$	33.55	40.68	434.11
${W_M}\left( t \right)$	780754.64	932521.90	–
${W_D}\left( t \right)$	311398.19	328912.79	–
${W_I}\left( t \right)$	231760.22	241442.29	–
${W_T}\left( t \right)$	1343913.05	1502876.98	11825814.85

Firstly, compared with the situation under decentralized decision-making, the collaborative innovation efforts and profit, the level of supply chain innovation, the level of domestic substitution and the total profit of the supply chain members in the centralized decision-making situation are significantly improved. This shows that when all members in the supply chain aim to maximise the total profit, it is beneficial to both the enterprises themselves and the supply chain as a whole.

Furthermore, compared with the two decentralized decision-making scenarios, the manufacturer’s process improvement effort and the designer’s innovation effort correspond equally, while the integrator’s compatibility effort increases by 66.67% in the cost-sharing scenario. In addition, after introducing the cost-sharing contract, the level of innovation in the supply chain remained the same, while the level of domestic substitution increased by 17.52%. The profits of the manufacturer, designer, and integrator increased by 16.257, 5.3255, and 4.01%, respectively, and the overall profit of the supply chain increased by 10.577%. This shows that when the designer (the leading company) chooses to stimulate its collaborative innovation by bearing part of the downstream integrator’s compatibility costs, it not only improves the integrator’s compatibility efforts and profits but also does not affect the designer’s collaborative innovation efforts and the innovation level of the supply chain. In addition, the profits of designers and manufacturers can be enhanced, thus making the total profit of the supply chain higher than when there is no cost-sharing contract. This is because the integrator’s compatibility efforts promote the level of domestic substitution, which benefits all firms in the supply chain.

**Fig. 2:**
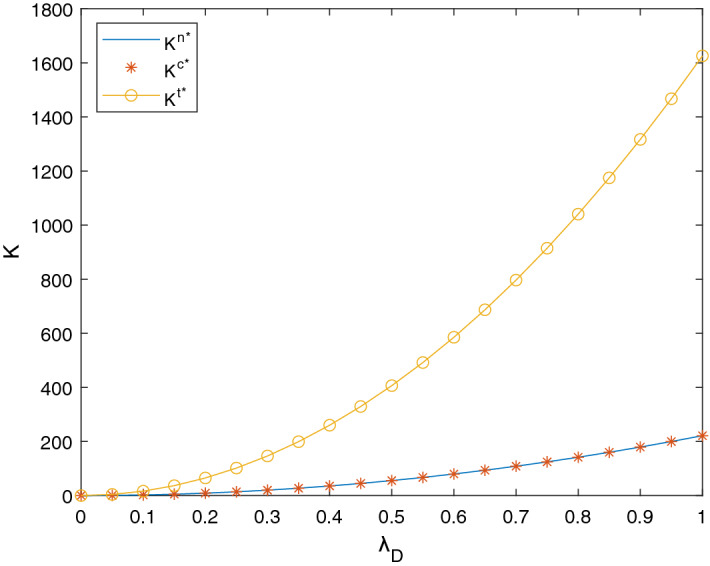
Influence of ${\mathrm{{\lambda }}_\mathrm{{D}}}$ on the level of innovation

**Fig. 3:**
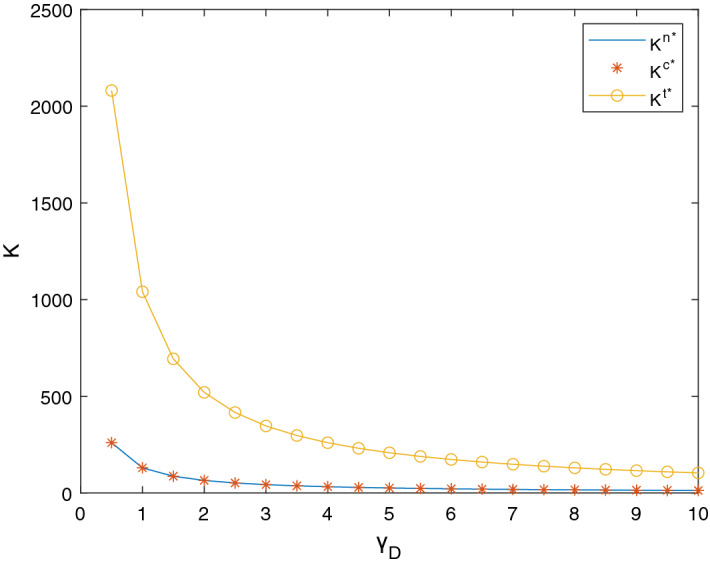
Influence of ${\gamma _D}$ on the level of innovation

**Fig. 4:**
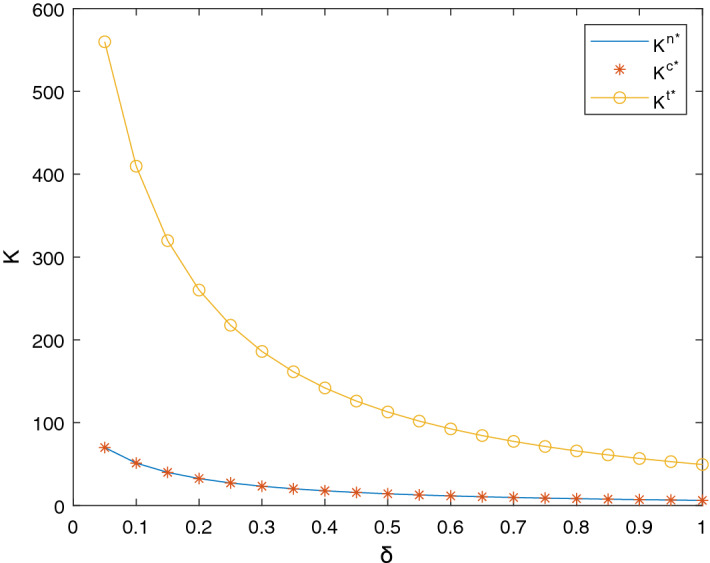
Influence of $\mathrm{{\delta }}$ on the level of innovation

Figures 2, 3 and 4 show how the level of supply chain innovation varies with the parameters ${\lambda _D}$, ${\gamma _D}$ and $\mathrm{{\delta }}$ for the three different scenarios. Figure 2 shows that at a given time t, the level of supply chain innovation varies with the coefficient of influence of the manufacturer’s innovation effort for all three scenarios, increasing with each. It can be seen from Fig. 2 that the trend of the level of supply chain innovation with the coefficient of influence of manufacturer innovation effort ${lambda _D}$ is consistent across the three scenarios when time t is specific, increasing with ${\lambda _D}$. The level of supply chain innovation under centralised decision-making is higher than that under the two decentralised decision-making scenarios. Furthermore, the level of innovation in the supply chain is equal under the two decentralised decisions because the designer’s innovation effort is equal under the two decentralised decisions. Whereas the innovation efforts entirely determine the innovation level of the monolithic machine in addition to other given parameters ${\mathrm{{E}}_D}\left( t \right)$.

As can be seen from Fig. 3, when the time t is certain, the innovation level in all three cases decreases with the designer’s innovation cost coefficient, indicating that the more difficult the MCU designer needs to overcome to carry out innovation, the poorer the collaborative innovation effect of upstream and downstream enterprises in the supply chain is.

From Fig. 4, it can be seen that the innovation level of the supply chain in all three cases decreases with the decline rate of technological innovation. Designers should therefore invest more innovation efforts to adapt to the ever-changing technology; otherwise, the effect of innovation will be significantly reduced.

**Fig. 5:**
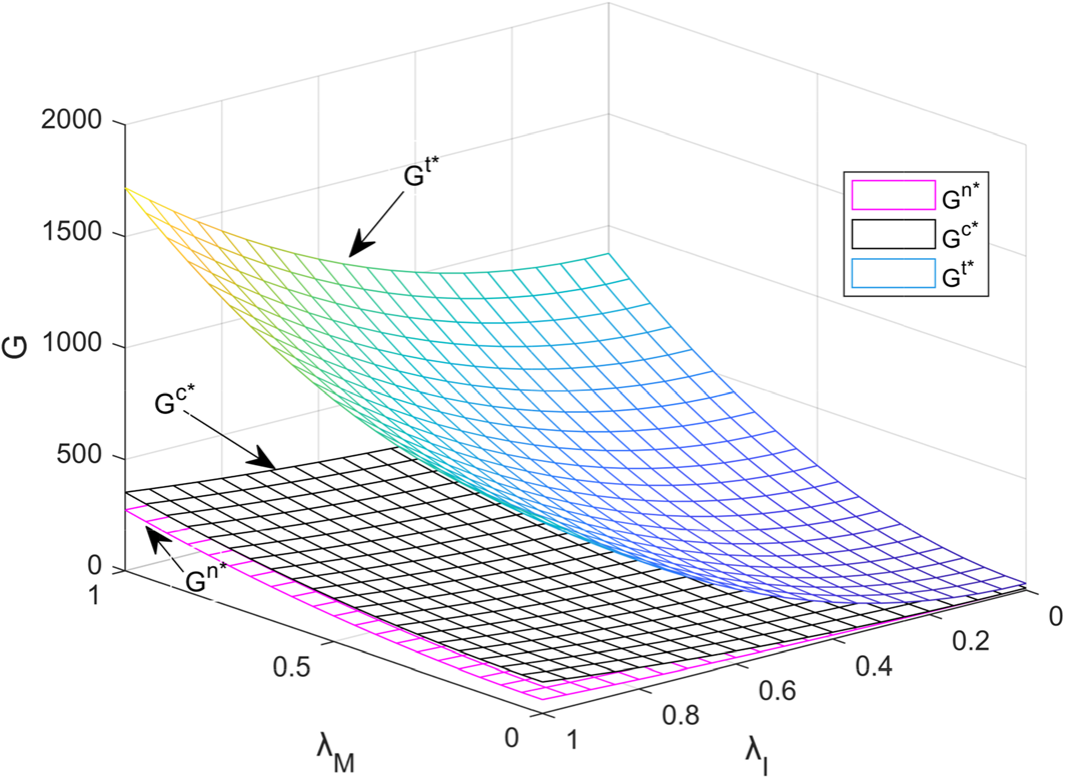
Influence of ${\mathrm{{\lambda }}_\mathrm{{I}}}$ and ${\mathrm{{\lambda }}_\mathrm{{M}}}$ on the level of domestic substitution

As shown in Fig. 5, the level of domestic substitution increases with the coefficient of influence coefficient of the manufacturer’s process improvement efforts and the integrator’s compatibility efforts on the domestic substitution level. This indicates that the more influential the role played by upstream and downstream enterprises in domestic substitution, the better the effect of domestic substitution.

**Fig. 6:**
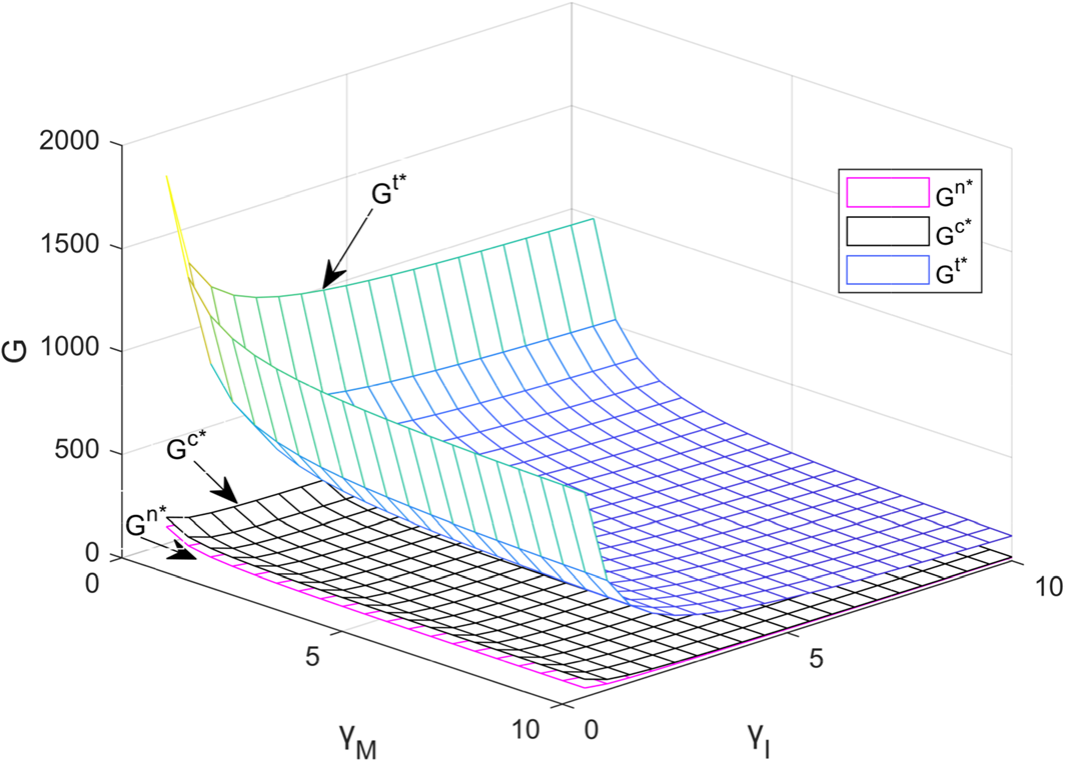
Influence of ${\gamma _M}$ and ${\gamma _I}$ on the level of domestic substitution

As seen from Fig. 6, the level of local substitution of MCUs decreases with ${\gamma _M}$ and ${\gamma _I}$, cost influence coefficient of the effort of the manufacturers and integrators involved in collaborative innovation. Therefore, if designers want to establish an independence ecosystem in China, they should fully consider the compatibility of downstream companies and reduce the resistance of downstream participation in collaborative innovation at the same time as performance innovation.

**Fig. 7:**
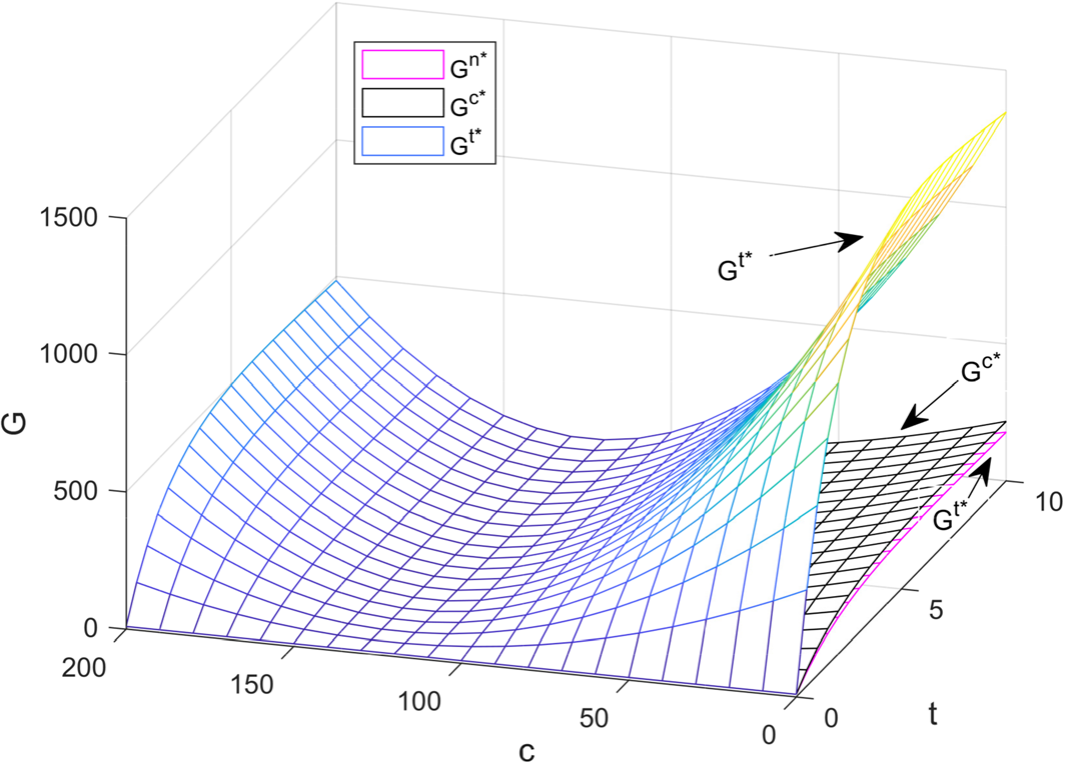
Influence of c and t on the level of domestic substitution

Figure 7 depicts the relationship between the domestic substitution level and MCU manufacturing cost and time. Under three different decision scenarios, the domestic substitution level of MCU increases with time and eventually plateaus, which indicates that long-term supply chain cooperation to promote the localization process is feasible; the growth of the domestic substitution level gradually slows down, which may be due to the decay rate of technological innovation and the local substitution process itself. With the increased chip manufacturing cost, the domestic substitution level will decrease and increase rapidly when the manufacturing cost reaches a certain threshold. In the current MCU market, chip manufacturing costs have increased exponentially due to the epidemic and other factors, and downstream integrators even face the dilemma that even a chip is difficult to obtain. However, from the numerical simulation results, the increase in MCU manufacturing cost may stimulate the innovation enthusiasm of domestic designers, thus promoting the process of domestic substitution. Although this conclusion is unconventional, it meets the actual MCU development situation in China. Therefore, the supply chain members should seize the current opportunity of MCU local substitution, meet the challenges brought by the epidemic and other factors, and actively participate in collaborative innovation.

**Fig. 8:**
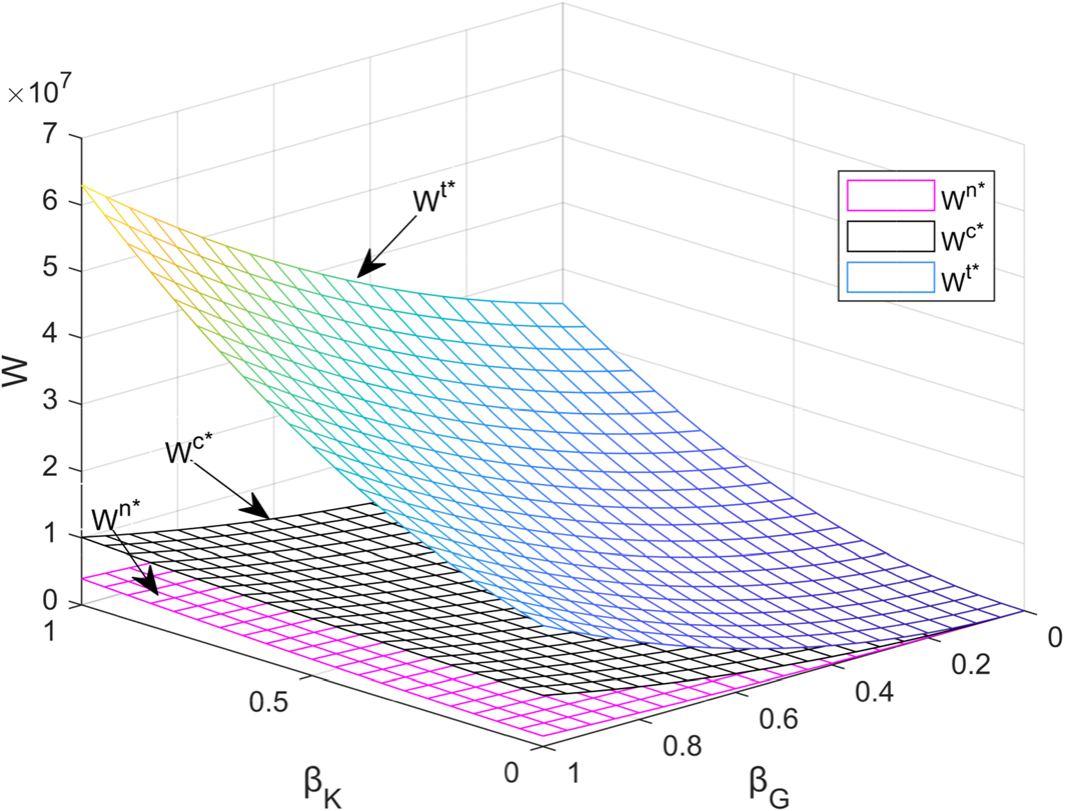
Influence of ${\beta _K}$ and ${\beta _G}$ on profit

Figure 8 shows how the total profit of the supply chain varies with the influence coefficient of innovation level and domestic substitution level on the market demand in the three scenarios. As the level of innovation and domestic substitution level on market demand increases, the supply chain’s total profit gradually increases, indicating that the end market’s attention to MCU local substitution innovation can promote the collaborative innovation of enterprises. Moreover, cost-sharing contracts can increase the supply chain’s total profit to some extent. Furthermore, the total profit of the supply chain is maximized in the case of cooperation mode.

**Fig. 9:**
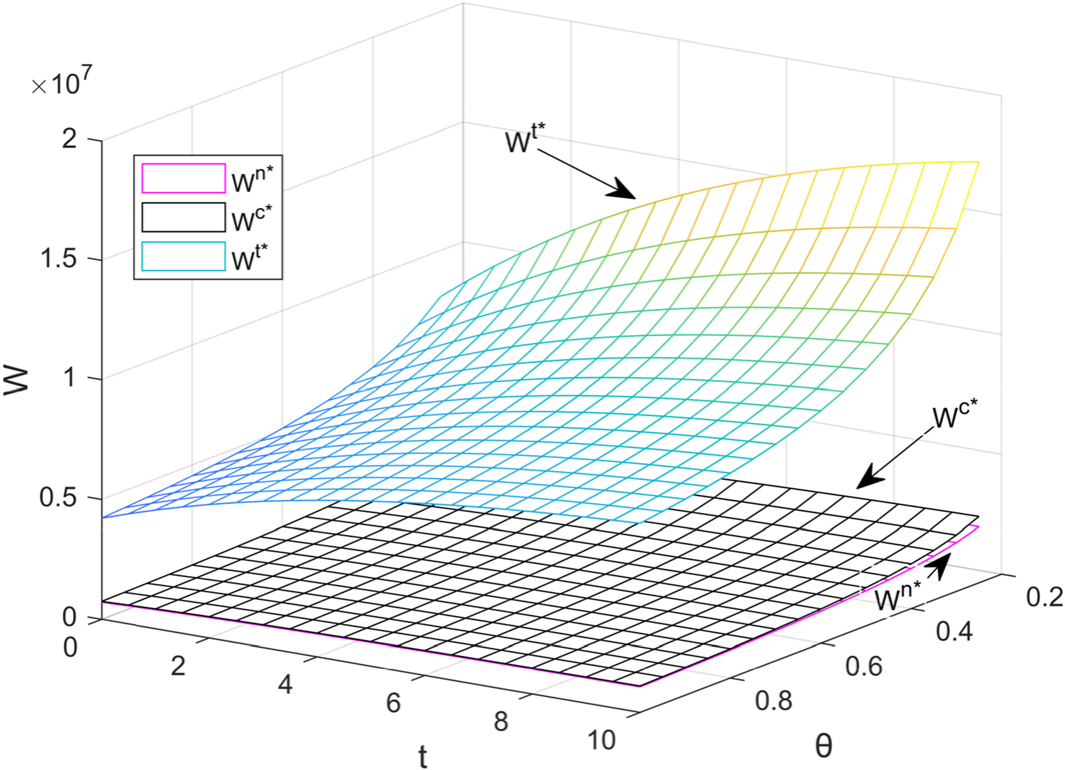
Influence of $\mathrm{{\theta }}$ and t on profit

Figure 9 depicts the variation of the total profit of the supply chain with time and the decline rate of the domestic substitution level. The total profit of the supply chain is highest in the case of centralized decision-making with the collaboration of supply chain members, indicating that the long-term collaborative innovation of supply chain members is conducive to the increase of total revenue of the supply chain. Suppose the domestic supply chain cannot replace and innovate advanced MCUs promptly. In that case, the market demand for domestic MCU will decrease, further affecting the total profit of the domestic MCU supply chain.

## Conclusions and Management Insights

### Conclusions

Previously, scholars used the traditional static game theory to study the supply chain collaborative innovation problem. However, the domestic replacement of integrated circuits in China is a long-term dynamic process. Therefore, this paper embeds the supply chain collaborative innovation theory into the IC domestic substitution scenario, introduces the time factor, and establishes a three-level MCU supply chain collaborative innovation dynamic game model, which fills the gap in the theoretical study of IC domestic substitution. This paper considers the two pain points of technological innovation and compatibility faced in the local replacement of MCU chips. It uses MCU’s innovation and domestic substitution levels as two state variables to study. A new supply chain coordination model, the "cost-sharing+two-part pricing" contract, is designed to provide a new theoretical reference for coordinating the strategic choices of IC supply chain members. Finally, numerical analysis is used to characterise the trends of innovation level, domestic substitution level and supply chain benefits in response to the influencing factors. Based on the above study, the following conclusions can be drawn:

1. In the case of centralised decision making, the optimal collaborative innovation input of supply chain members, the level of supply chain innovation, the level of domestic substitution and the total profit of the supply chain all achieve better results than in the case of decentralized decision making.
2. Under a design-led cost-sharing scenario, the integrator’s compatibility effort, market demand, level of domestic substitution and supply chain profits are all increased. The designer’s innovation efforts and MCU innovation levels are not affected. This is because the level of product innovation is only influenced by the designer’s innovation effort and the rate of technology decline, independent of other factors.
3. A designer-led two-part pricing + cost-sharing contract allows the integrator to achieve the level of effort that would be expected under centralised decision-making, thereby increasing the profitability of each supply chain member and achieving Pareto improvements.
4. With the increase of manufacturing cost, the MCU domestic substitution level trend with the change of cost is "U" shape, that is, first decreases and then gradually increases. In today’s global "core shortage", chip manufacturing costs are increasing exponentially, which may catalyse the domestic replacement of Chinese MCUs. This may be good news for China, where the IC industry is less developed than in Europe and the US. Chinese MCU start-ups should seize the opportunity to break through the core technology bottleneck and gradually promote the domestic replacement of MCUs.

### Management Insights

A counterintuitive finding of this study is that the level of domestic substitution and supply chain profits decay briefly and then gradually increase as manufacturing costs increase. Moreover, over time, the innovation level, domestic substitution level, and all parties’ profits increase steadily, with the growth trend levelling off. Therefore, although the prices of rare gases and semiconductor materials required to manufacture chips are soaring for MCU manufacturers, it is a good time for manufacturers to expand capacity and increase profits. Manufacturers can seize the opportunity to work with domestic designers to meet the challenges of rising costs due to the lack of chips.

For MCU designers, as the technology in the IC industry is changing rapidly, they must design a reasonable supply chain coordination contract to actively lead the supply chain to collaborate and innovate, in addition to continuous R &D and technology enhancement. This is the only way to minimise the impact of *supply disruptions* and *capacity declines* on the industry.

For integrators, although using replacement MCUs will inevitably lead to higher costs due to compatibility issues, in the long run, participation in the collaborative innovation of the local supply chain will increase the integrator’s profitability and help to improve the overall supply chain efficiency. Integrators can actively participate in designing and signing supply chain contracts to ensure optimal integrator strategies drive domestic substitution.

### Research Perspectives

As the world’s leading countries compete increasingly in high technology, more and more countries realise that an autonomous and controllable IC supply chain is critical to ensure national security and stable economic development. In order to cope with the ever-changing technology in the IC industry, MCU designers can only reduce the impact of external *supply disruption* and *capacity drop* on the industry development by continuously strengthening technology, improving innovation, actively guiding the collaborative innovation of the supply chain, encouraging upstream and downstream enterprises to participate in the domestic replacement actively, and building a domestic ecosystem. In order to reduce the impact on the development of the industry caused by external factors such as *out-of-supply* and *declining production capacity*, this paper focuses on building a three-stage domestic MCU ecosystem. In this paper, we study the strategy and incentive mechanism of collaborative innovation within the supply chain under the dynamic structure by constructing a differential game model for the three-level supply chain of domestic MCUs. However, there are still some limitations in this study.

Firstly, this paper introduces the innovation and compatibility costs faced by MCU, a typical component of ICs, in the domestic substitution into the differential game model and links it to the level of innovation and the level of local substitution, portraying the dynamic change process of the level of innovation and the level of local substitution. In practice, apart from MCUs, components such as CPUs, GPUs and EDA design software in the IC industry are also in urgent need of domestic substitution. The situation is even more complex regarding this process’s coordination and development of the software and hardware ecology. Therefore, when studying the local replacement of software in the future, in addition to the common issues such as innovation and compatibility mentioned in this paper, it can also be carried out from the perspective of ecosystem construction.

Second, in recent years, governments have actively developed incentive and subsidy strategies to encourage independent innovation and R&D in IC companies. This paper does not consider the role of government subsidies in domestic substitution. With the increasing competition among the world’s major countries in the field of high technology, more and more countries realise that an autonomous and controllable IC supply chain is essential to ensure national security and smooth economic development. In the follow-up study, government subsidy policies can be studied and discussed.

## Data Availability

Not applicable.

## Proof for Corollary 1

In all three cases, ${{E}_{D}}{{^{n}}^{*}}$, ${{E}_{I}}^{*}$, ${{E}_{M}}^{*}$, ${{K}_{\infty }}^{*}$ and ${{G}_{\infty }}^{*}$follows the same trend with the relevant parameters. Therefore, we take the case under decentralised decision-making without cost-sharing as an example. Taking the first derivatives of ${{E}_{D}}{{^{n}}^{*}}$ with respect to $\text { }\!\!\alpha \!\!\text { }$ , $\rho$ , $\text { }\!\!\delta \!\!\text { }$ , $\text { }\!\!\theta \!\!\text { }$ , ${{\beta }_{P}}$ , ${{\beta }_{K}}$ , ${{\beta }_{G}}$ , ${{\gamma }_{M}}$ , ${{\gamma }_{D}}$ , ${{\gamma }_{I}}$ , ${{\lambda }_{M}}$ , ${{\lambda }_{D}}$ , ${{\lambda }_{I}}~$and $\text { }\!\!\varepsilon \!\!\text { }\!\!~\!\!\text { }$, we can obtain the result in the first row of Table 1:

$$\frac{\partial E{{_{D}^{n}}^{*}}}{\partial \alpha }=\frac{\left( \alpha -{{\beta }_{P}}c \right) \left[ {{\beta }_{k}}\left( \rho +\theta \right) +{{\beta }_{G}}\varepsilon \right] {{\lambda }_{D}}}{16{{\beta }_{P}}\left( \rho +\delta \right) \left( \rho +\theta \right) {{\gamma }_{D}}}>0$$,

$$\frac{\partial E{{_{D}^{n}}^{*}}}{\partial \rho }=\frac{-{{\left( \alpha -{{\beta }_{P}}c \right) }^{2}}\left[ {{\beta }_{K}}{{\left( \rho +\theta \right) }^{2}}+{{\beta }_{G}}(2\rho +\theta +\delta ) \right] {{\lambda }_{D}}}{32{{\beta }_{P}}{{\left( \rho +\delta \right) }^{2}}{{\left( \rho +\theta \right) }^{2}}{{\gamma }_{D}}}<0$$,

$$\frac{\partial E{{_{D}^{n}}^{*}}}{\partial \delta }=\frac{-{{\left( \alpha -{{\beta }_{P}}c \right) }^{2}}\left[ {{\beta }_{K}}\left( \rho +\theta \right) +{{\beta }_{G}}\varepsilon \right] {{\lambda }_{D}}}{32{{\beta }_{P}}{{\left( \rho +\delta \right) }^{2}}\left( \rho +\theta \right) {{\gamma }_{D}}}<0$$,

$$\frac{\partial E{{_{D}^{n}}^{*}}}{\partial \theta }=\frac{-{{\left( \alpha -{{\beta }_{P}}c \right) }^{2}}{{\beta }_{G}}\varepsilon {{\lambda }_{D}}}{32{{\beta }_{P}}\left( \rho +\delta \right) {{\left( \rho +\theta \right) }^{2}}{{\gamma }_{D}}}<0$$,

$$\frac{\partial E{{_{D}^{n}}^{*}}}{\partial {{\beta }_{P}}}=\frac{-(\alpha -{{\beta }_{P}}c)(2{{\beta }_{P}}+1)\left[ {{\beta }_{K}}\left( \rho +\theta \right) +{{\beta }_{G}}\varepsilon \right] {{\lambda }_{D}}}{32{{\beta }_{P}}^{2}\left( \rho +\delta \right) \left( \rho +\theta \right) {{\gamma }_{D}}}<0$$,

$$\frac{\partial E{{_{D}^{n}}^{*}}}{\partial {{\beta }_{K}}}=\frac{{{(\alpha -{{\beta }_{P}}c)}^{2}}{{\lambda }_{D}}}{32{{\beta }_{P}}\left( \rho +\delta \right) {{\gamma }_{D}}}>0$$,

$$\frac{\partial E{{_{D}^{n}}^{*}}}{\partial {{\beta }_{G}}}=\frac{{{(\alpha -{{\beta }_{P}}c)}^{2}}{{\lambda }_{D}}\varepsilon }{32{{\beta }_{P}}\left( \rho +\theta \right) \left( \rho +\delta \right) {{\gamma }_{D}}}>0$$,

$$\frac{\partial E{{_{D}^{n}}^{*}}}{\partial {{\gamma }_{D}}}=\frac{-{{(\alpha -{{\beta }_{P}}c)}^{2}}\left[ {{\beta }_{K}}\left( \rho +\theta \right) +{{\beta }_{G}}\varepsilon \right] {{\lambda }_{D}}}{32{{\beta }_{P}}\left( \rho +\theta \right) \left( \rho +\delta \right) {{\gamma }_{D}}^{2}}<0$$,

$$\frac{\partial E{{_{D}^{n}}^{*}}}{\partial \varepsilon }=\frac{{{(\alpha -{{\beta }_{P}}c)}^{2}}{{\beta }_{G}}{{\lambda }_{D}}}{32{{\beta }_{P}}\left( \rho +\theta \right) \left( \rho +\delta \right) {{\gamma }_{D}}}>0$$.

The world is currently in an extreme shortage of chip manufacturing capacity, and downstream integrators are willing to pay ten or even a hundred times higher than the original price to purchase chips. At this point, the MCU market demand is much greater than the price sensitivity factor. Therefore, we only consider $\alpha -{{\beta }_{P}}c>0$ to ensure that our conclusions are realistic.

The proof for the remainder of the table follows the same process as above and is not repeated here. Then, we can obtain the conclusions of Corollary 1.

## Proof for Colollary 2

$$  {\text{E}}_{D}^{{n*}}  = {\text{E}}_{D}^{{c*}} ,{\text{E}}_{D}^{{t*}}  - {\text{E}}_{D}^{{c*}}  = \frac{{{\text{ }}\Delta _{4} [\beta _{K} \left( {\rho  + \theta } \right) + \beta _{G} \varepsilon ]\lambda _{D} }}{{\gamma _{D} \left( {\rho  + \delta } \right)\left( {\rho  + \theta } \right)}} - \frac{{\Delta _{2} \left[ {\beta _{{\text{k}}} \left( {\rho  + \theta } \right) + {\text{ }}\beta _{{\text{G}}} \varepsilon } \right]\lambda _{D} }}{{\left( {\rho  + \delta } \right)\left( {\rho  + \theta } \right)\gamma _{D} }} = \frac{{\left( {\Delta _{4}  - {\text{ }}\Delta _{2} } \right)[(\beta _{K} \left( {\rho  + \theta } \right) + \beta _{G} \varepsilon ]\lambda _{D} }}{{\gamma _{D} \left( {\rho  + \delta } \right)\left( {\rho  + \theta } \right)}}{\text{  > }}0  $$

Then we can conclude $\text {E}{{_{D}^{n}}^{*}}=\text {E}{{_{D}^{c}}^{*}}<\text {E}{{_{D}^{t}}^{*}}$.

${{\text {K}}^{n}}^{*}\text {=}{{\text {K}}^{c}}^{*}$, ${{\text {K}}^{t}}^{*}-{{\text {K}}^{c}}^{*}=\frac{\left( {{\text { }\!\!\Delta \!\!\text { }}_{4}}-{{\text { }\!\!\Delta \!\!\text { }}_{2}} \right) [\left( {{\beta }_{K}}\left( \rho +\theta \right) +{{\beta }_{G}}\varepsilon \right] {{\lambda }_{D}}^{2}}{{{\gamma }_{D}}\left( \rho +\delta \right) \left( \rho +\theta \right) }\left( 1-{{e}^{-\delta t}} \right) >0$. Then we can conclude ${{\text {K}}^{n}}^{*}={{\text {K}}^{c}}^{*}<{{\text {K}}^{t}}^{*}$. Based on the above analysis, Colollary 2 can be obtained.

## Proof for Colollary 3

$$\text {E}{{_{M}^{n}}^{*}}=\text {E}{{_{M}^{c}}^{*}}$$, $$E{{_{M}^{t}}^{*}}-E{{_{M}^{c}}^{*}}=\frac{{{\Delta }_{4}}{{\beta }_{G}}{{\lambda }_{M}}}{\left( \rho +\theta \right) {{\gamma }_{M}}}-\frac{{{\Delta }_{1}}{{\beta }_{G}}{{\lambda }_{M}}}{\left( \rho +\theta \right) {{\gamma }_{M}}}=\frac{{{\beta }_{G}}({{\Delta }_{4}}-{{\Delta }_{1}}){{\lambda }_{M}}}{{{\gamma }_{M}}\left( \rho +\theta \right) }=\frac{3{{\left( \alpha -{{\beta }_{P}}c \right) }^{2}}{{\beta }_{G}}{{\lambda }_{M}}}{16{{\beta }_{P}}{{\gamma }_{I}}\left( \rho +\theta \right) }>0$$.

Then we can conclude $\text {E}{{_{M}^{n}}^{*}}=\text {E}{{_{M}^{c}}^{*}}<\text {E}{{_{M}^{t}}^{*}}$.

$$\text {E}{{_{I}^{c}}^{*}}-\text {E}{{_{I}^{n}}^{*}}=\frac{{{\text { }\!\!\beta \!\!\text { }}_{\text {G}}}\left( 2{{\text { }\!\!\Delta \!\!\text { }}_{3}}+{{\text { }\!\!\Delta \!\!\text { }}_{1}} \right) {{\lambda }_{I}}}{2{{\gamma }_{I}}\left( \rho +\theta \right) }-\frac{{{\text { }\!\!\Delta \!\!\text { }}_{3}}{{\text { }\!\!\beta \!\!\text { }}_{\text {G}}}{{\lambda }_{I}}}{\left( \rho +\theta \right) {{\gamma }_{I}}}=\frac{{{\text { }\!\!\beta \!\!\text { }}_{\text {G}}}{{\text { }\!\!\Delta \!\!\text { }}_{1}}{{\lambda }_{I}}}{2{{\gamma }_{I}}\left( \rho +\theta \right) }=\frac{{{\left( \text { }\!\!\alpha \!\!\text { }-{{\text { }\!\!\beta \!\!\text { }}_{\text {P}}}\text {c} \right) }^{2}}{{\text { }\!\!\beta \!\!\text { }}_{\text {G}}}}{32{{\text { }\!\!\beta \!\!\text { }}_{\text {P}}}{{\gamma }_{I}}\left( \rho +\theta \right) }>0$$,

$$\text {E}{{_{I}^{t}}^{*}}-\text {E}{{_{I}^{c}}^{*}}=\frac{{{\text { }\!\!\Delta \!\!\text { }}_{4}}{{\text { }\!\!\beta \!\!\text { }}_{\text {G}}}{{\lambda }_{I}}}{\left( \rho +\theta \right) {{\gamma }_{I}}}-\frac{{{\text { }\!\!\beta \!\!\text { }}_{\text {G}}}\left( 2{{\text { }\!\!\Delta \!\!\text { }}_{3}}+{{\text { }\!\!\Delta \!\!\text { }}_{1}} \right) }{2{{\gamma }_{I}}\left( \rho +\theta \right) }=\frac{{{\text { }\!\!\beta \!\!\text { }}_{\text {G}}}\text {(2}{{\text { }\!\!\Delta \!\!\text { }}_{4}}-2{{\text { }\!\!\Delta \!\!\text { }}_{3}}-{{\text { }\!\!\Delta \!\!\text { }}_{1}}\text {)}{{\text { }\!\!\Delta \!\!\text { }}_{1}}{{\lambda }_{I}}}{2{{\gamma }_{I}}\left( \rho +\theta \right) }=\frac{13{{\left( \text { }\!\!\alpha \!\!\text { }-{{\text { }\!\!\beta \!\!\text { }}_{\text {P}}}\text {c} \right) }^{2}}{{\text { }\!\!\beta \!\!\text { }}_{\text {G}}}{{\lambda }_{I}}}{32{{\text { }\!\!\beta \!\!\text { }}_{\text {P}}}{{\gamma }_{I}}\left( \rho +\theta \right) }>0$$.

Then we can conclude $\text {E}{{_{I}^{n}}^{*}}<\text {E}{{_{I}^{c}}^{*}}<\text {E}{{_{I}^{t}}^{*}}.$

${\mathrm{{G}}^c}^* - {\mathrm{{G}}^n}^* = \left( {1 - {e^{ - \theta t}}} \right) \left[ {\mathrm{{G}}_\infty ^c - \mathrm{{G}}_\infty ^n + \frac{\varepsilon }{{\theta + \delta }}\left( {\mathrm{{K}}_\infty ^c - \mathrm{{K}}_\infty ^n} \right) } \right] = \left( {1 - {e^{ - \theta t}}} \right) \frac{{{\mathrm{{\beta }}_\mathrm{{G}}}\left( {2{\mathrm{{\Delta }}_3} - {\mathrm{{\Delta }}_1}} \right) {\lambda _I}^2}}{{2\theta {\gamma _I}\left( {\rho + \theta } \right) }} > 0$, that is ${\mathrm{{G}}^c}^* > {\mathrm{{G}}^n}^*$. ${\mathrm{{G}}^t}^* > {\mathrm{{G}}^c}^*$ can be proved in the same way.

Then we can conclude ${{\text {G}}^{n}}^{*}={{\text {G}}^{c}}^{*}<{{\text {G}}^{t}}^{*}$.

Based on the above analysis, Colollary 3 can be obtained.

## Proof for Corollary 4

$$\text {W}{{_{M}^{c}}^{\text {*}}}-\text {W}{{_{M}^{n}}^{\text {*}}}=\frac{{{\text { }\!\!\Delta \!\!\text { }}_{1}}[{{\text { }\!\!\beta \!\!\text { }}_{\text {k}}}\left( \rho +\theta \right) +{{\text { }\!\!\beta \!\!\text { }}_{\text {G}}}\varepsilon ]}{\left( \rho +\delta \right) \left( \rho +\theta \right) }\left( {{K}^{c}}^{*}-{{K}^{n}}^{*} \right) +\frac{{{\text { }\!\!\Delta \!\!\text { }}_{1}}{{\text { }\!\!\beta \!\!\text { }}_{\text {G}}}}{\left( \rho +\theta \right) }({{G}^{c}}^{*}-{{G}^{n}}^{*})+\frac{{{\text { }\!\!\Delta \!\!\text { }}_{1}}\left( 2{{\text { }\!\!\Delta \!\!\text { }}_{2}}-{{\text { }\!\!\Delta \!\!\text { }}_{3}} \right) {{\text { }\!\!\beta \!\!\text { }}_{\text {G}}}^{2}{{\lambda }_{I}}^{2}}{\rho {{\gamma }_{I}}{{\left( \rho +\theta \right) }^{2}}}=\frac{{{\text { }\!\!\Delta \!\!\text { }}_{1}}[{{\text { }\!\!\beta \!\!\text { }}_{\text {k}}}\left( \rho +\theta \right) +{{\text { }\!\!\beta \!\!\text { }}_{\text {G}}}\varepsilon ]}{\left( \rho +\delta \right) \left( \rho +\theta \right) }\left( {{K}^{c}}^{*}-{{K}^{n}}^{*} \right) +\frac{{{\text { }\!\!\Delta \!\!\text { }}_{1}}{{\text { }\!\!\beta \!\!\text { }}_{\text {G}}}}{\left( \rho +\theta \right) }({{G}^{c}}^{*}-{{G}^{n}}^{*})+\frac{3{{\text { }\!\!\beta \!\!\text { }}_{\text {G}}}^{2}{{\left( \text { }\!\!\alpha \!\!\text { }-{{\text { }\!\!\beta \!\!\text { }}_{\text {P}}}\text {c} \right) }^{2}}{{\lambda }_{I}}^{2}}{1024\rho {{\gamma }_{I}}{{\left( \rho +\theta \right) }^{2}}{{\text { }\!\!\beta \!\!\text { }}_{P}}^{2}}>0$$, that is $$\text {W}{{_{M}^{n}}^{*}}<\text {W}{{_{M}^{c}}^{*}}$$.

$$\text {W}{{_{D}^{c}}^{\text {*}}}-\text {W}{{_{D}^{n}}^{\text {*}}}=\frac{{{\text { }\!\!\Delta \!\!\text { }}_{2}}[{{\text { }\!\!\beta \!\!\text { }}_{\text {k}}}\left( \rho +\theta \right) +{{\text { }\!\!\beta \!\!\text { }}_{\text {G}}}\varepsilon ]}{\left( \rho +\delta \right) \left( \rho +\theta \right) }\left( {{K}^{c}}^{*}-{{K}^{n}}^{*} \right) +\frac{{{\text { }\!\!\Delta \!\!\text { }}_{2}}{{\text { }\!\!\beta \!\!\text { }}_{\text {G}}}}{\left( \rho +\theta \right) }({{G}^{c}}^{*}-{{G}^{n}}^{*})+\frac{{{\left( 2{{\text { }\!\!\Delta \!\!\text { }}_{2}}-{{\text { }\!\!\Delta \!\!\text { }}_{3}} \right) }^{2}}{{\text { }\!\!\beta \!\!\text { }}_{\text {G}}}^{2}{{\lambda }_{I}}^{2}}{\rho {{\gamma }_{I}}{{\left( \rho +\theta \right) }^{2}}}>0$$, that is $$\text {W}{{_{D}^{n}}^{*}}<\text {W}{{_{D}^{c}}^{*}}$$.

$$\text {W}{{_{I}^{c}}^{\text {*}}}-\text {W}{{_{I}^{n}}^{\text {*}}}=\frac{{{\text { }\!\!\Delta \!\!\text { }}_{3}}[{{\text { }\!\!\beta \!\!\text { }}_{\text {k}}}\left( \rho +\theta \right) +{{\text { }\!\!\beta \!\!\text { }}_{\text {G}}}\varepsilon ]}{\left( \rho +\delta \right) \left( \rho +\theta \right) }\left( {{K}^{c}}^{*}-{{K}^{n}}^{*} \right) +\frac{{{\text { }\!\!\Delta \!\!\text { }}_{3}}{{\text { }\!\!\beta \!\!\text { }}_{\text {G}}}}{\left( \rho +\theta \right) }({{G}^{c}}^{*}-{{G}^{n}}^{*})+\frac{{{\text { }\!\!\Delta \!\!\text { }}_{3}}\left( 2{{\text { }\!\!\Delta \!\!\text { }}_{2}}-{{\text { }\!\!\Delta \!\!\text { }}_{3}} \right) {{\text { }\!\!\beta \!\!\text { }}_{\text {G}}}^{2}{{\lambda }_{I}}^{2}}{\rho {{\gamma }_{I}}{{\left( \rho +\theta \right) }^{2}}}=\frac{{{\text { }\!\!\Delta \!\!\text { }}_{3}}[{{\text { }\!\!\beta \!\!\text { }}_{\text {k}}}\left( \rho +\theta \right) +{{\text { }\!\!\beta \!\!\text { }}_{\text {G}}}\varepsilon ]}{\left( \rho +\delta \right) \left( \rho +\theta \right) }\left( {{K}^{c}}^{*}-{{K}^{n}}^{*} \right) +\frac{{{\text { }\!\!\Delta \!\!\text { }}_{3}}{{\text { }\!\!\beta \!\!\text { }}_{\text {G}}}}{\left( \rho +\theta \right) }({{G}^{c}}^{*}-{{G}^{n}}^{*})+\frac{3{{\left( \text { }\!\!\alpha \!\!\text { }-{{\text { }\!\!\beta \!\!\text { }}_{\text {P}}}\text {c} \right) }^{2}}{{\text { }\!\!\beta \!\!\text { }}_{\text {G}}}^{2}{{\lambda }_{I}}^{2}}{128\rho {{\gamma }_{I}}{{\left( \rho +\theta \right) }^{2}}{{\text { }\!\!\beta \!\!\text { }}_{P}}^{2}}>0$$, that is $$\text {W}{{_{I}^{n}}^{*}}<\text {W}{{_{I}^{c}}^{*}}$$.

$$\text {W}{{_{T}^{t}}^{*}}-(\text {W}{{_{M}^{c}}^{*}}+\text {W}{{_{D}^{c}}^{*}}+\text {W}{{_{I}^{c}}^{*}})=\frac{\left( {{\text { }\!\!\Delta \!\!\text { }}_{2}}+{{\text { }\!\!\Delta \!\!\text { }}_{3}}+{{\text { }\!\!\Delta \!\!\text { }}_{1}} \right) [{{\text { }\!\!\beta \!\!\text { }}_{\text {k}}}\left( \rho +\theta \right) +{{\text { }\!\!\beta \!\!\text { }}_{\text {G}}}\varepsilon ]}{\left( \rho +\delta \right) \left( \rho +\theta \right) }({{K}^{c}}^{*}-{{K}^{n}}^{*})+\frac{\left( {{\text { }\!\!\Delta \!\!\text { }}_{2}}+{{\text { }\!\!\Delta \!\!\text { }}_{3}}+{{\text { }\!\!\Delta \!\!\text { }}_{1}} \right) {{\text { }\!\!\beta \!\!\text { }}_{\text {G}}}}{\left( \rho +\theta \right) }({{G}^{c}}^{*}-{{G}^{n}}^{*})+\frac{1}{\rho }\left[ \frac{\left( 16{{\text { }\!\!\Delta \!\!\text { }}_{2}}{{\text { }\!\!\Delta \!\!\text { }}_{3}}-4{{\text { }\!\!\Delta \!\!\text { }}_{1}}{{\text { }\!\!\Delta \!\!\text { }}_{3}}+4{{\text { }\!\!\Delta \!\!\text { }}_{3}}^{2}-8{{\text { }\!\!\Delta \!\!\text { }}_{1}}{{\text { }\!\!\Delta \!\!\text { }}_{2}}-3{{\text { }\!\!\Delta \!\!\text { }}_{1}}^{2} \right) {{\text { }\!\!\beta \!\!\text { }}_{\text {G}}}^{2}{{\lambda }_{I}}^{2}}{8{{\gamma }_{I}}{{\left( \rho +\theta \right) }^{2}}} \right] >0 $$, that is $$\text {W}{{_{M}^{c}}^{*}}+\text {W}{{_{D}^{c}}^{*}}+\text {W}{{_{I}^{c}}^{*}}<\text {W}{{_{T}^{t}}^{*}}$$.

$\text {W}{{_{M}^{n}}^{*}}+\text {W}{{_{D}^{n}}^{*}}+\text {W}{{_{I}^{n}}^{*}}<\text {W}{{_{M}^{c}}^{*}}+\text {W}{{_{D}^{c}}^{*}}+\text {W}{{_{I}^{c}}^{*}}$ can be proved in the same way.

Based on the above analysis, Colollary 4 can be obtained.
